# Construction of predictive promoter models on the example of antibacterial response of human epithelial cells

**DOI:** 10.1186/1742-4682-2-2

**Published:** 2005-01-12

**Authors:** Ekaterina Shelest, Edgar Wingender

**Affiliations:** 1 Dept. of Bioinformatics, UKG, University of Göttingen, Goldschmidtstr. 1, D-37077 Göttingen, Germany; 2 BIOBASE GmbH, Halchtersche Str. 33, D-38304 Wolfenbüttel, Germany

## Abstract

**Background:**

Binding of a bacteria to a eukaryotic cell triggers a complex network of interactions in and between both cells. *P. aeruginosa *is a pathogen that causes acute and chronic lung infections by interacting with the pulmonary epithelial cells. We use this example for examining the ways of triggering the response of the eukaryotic cell(s), leading us to a better understanding of the details of the inflammatory process in general.

**Results:**

Considering a set of genes co-expressed during the antibacterial response of human lung epithelial cells, we constructed a promoter model for the search of additional target genes potentially involved in the same cell response. The model construction is based on the consideration of pair-wise combinations of transcription factor binding sites (TFBS).

It has been shown that the antibacterial response of human epithelial cells is triggered by at least two distinct pathways. We therefore supposed that there are two subsets of promoters activated by each of them. Optimally, they should be "complementary" in the sense of appearing in complementary subsets of the (+)-training set. We developed the concept of complementary pairs, i.e., two mutually exclusive pairs of TFBS, each of which should be found in one of the two complementary subsets.

**Conclusions:**

We suggest a simple, but exhaustive method for searching for TFBS pairs which characterize the whole (+)-training set, as well as for complementary pairs. Applying this method, we came up with a promoter model of antibacterial response genes that consists of one TFBS pair which should be found in the whole training set and four complementary pairs.

We applied this model to screening of 13,000 upstream regions of human genes and identified 430 new target genes which are potentially involved in antibacterial defense mechanisms.

## Background

Promoter model construction is a way to utilize information about coexpressed genes; this kind of information becomes more and more available with the advent of gene expression mass data, mainly from microarray experiments. Having a promoter model at hand, one has (i) an explanatory model that and how the coexpressed gene may be coregulated, and (ii) a means to scan the whole genome for additional genes that may belong to the same "regulon". The field of searching for regulatory elements *in silico *and promoter modeling is already well-cultivated. In spite of numerous sophisticated approaches devoted to this subject [1-9], we still lack a standard method which would enable us to produce promoter models. This may indicate that the existing approaches have their distinct shortcomings and that, thus, the field is still open for new ideas.

The biological system we consider in this work is the transcriptional regulation of the response of lung epithelial cells to infection with *Pseudomonas aeruginosa*. Binding of bacteria to a eukaryotic cell triggers a complex network of interactions within and between both cells. *P. aeruginosa *is a pathogen that causes acute and chronic lung infections affecting pulmonary epithelial cells [10,11]. We use this example for examining the ways in which the response of the eukaryotic cell(s) is triggered, leading us to a better understanding of the details of the inflammatory process in general.

After adhesion of *P. aeruginosa *to the epithelial cells, the response of these cells is triggered by at least two distinct agents: bacterial lipopolysaccharides [12] and/or bacterial pilins or flaggelins [13]. Both pathways lead to the activation of the transcription factor NF-κB. It has also been shown that transcription factors AP-1 and C/EBP participate in this response [14,15]; pronounced hints on the participation of Elk-1 [16] have been reported as well. However, it is a commonly accepted view that transcription factors which are involved in a certain cellular response cooperate and in most cases act in a synergistic manner. Therefore, their binding sites are organized in a non-random manner [2,3,8,9].

We use this consideration as a basis for constructing a predictive promoter model. We searched for combinations of potential transcription factor binding sites (TFBS), considering those transcription factors (TFs) that are known to be involved in antibacterial responses. Some of the found combinations could be predicted from the fact that they may constitute well-known composite elements, like those containing NF-κB and C/EBP or NF-κB and Sp1 binding sites [TRANSCompel, [17]]. We start with a search for pairwise combinations of TFBS in a set of human genes published to be induced during antibacterial response, considering that combinations of the higher orders can be constructed from them later on.

We suggest a simple, but exhaustive method for searching for TFBS pairs which characterize the whole training set, and combinations of mutually exclusive pairs (complementary pairs). The idea of starting the analysis with a "seed" of sequences allows a very biology-driven way of initial filtering of information.To enhance the statistical reliability and to get additional evidence in TFBS combination search, we applied the principal idea of phylogenetic footprinting (using orthologous mouse promoters), yet proposing a different view on applicability of this approach.

Finally we came up with a promoter model which we applied to screening of 13,000 upstream regions human genes. We identified 430 new target genes which are potentially involved in antibacterial defense mechanisms.

## Results

### Development of the approach

In every step of our investigations we tried to combine purely computational approaches with the preexisting experiment-based knowledge, as it is represented in corresponding databases and literature, and with our own biological expertise. To develop a promoter model, the first task is to select those transcription factors, the binding sites of which shall consitute the model. The overwhelming majority of methods and tools estimating the relevance of predicted TF binding sites in promoter regions are based on their over- and underrepresentation in a positive (+) training set in comparison with some negative (-) training set. If, however, a binding site is ubiquitous, or very degenerate, so that it can be found frequently in any sequence, the comparison with basically any (-)-training would not reveal any significance for its occurrence. That tells nothing about their functionality in any specific case, which may be dependent on some additional factors and/or other conditions. Therefore, basing the decision about the relevance of a transcription factor for a certain cellular response solely on whether its predicted binding sites are overrepresented in the responding promoters may lead to a loss of important information. Thus, we did not rely on this kind of evidence but rather chose the candidate transcription factors according to available experimental data. We found 5 factors reported in literature as taking part in anti-bacterial or similar responses and selected them as candidate TFs [11,12,15,18-29]. Not all of these candidate TFs are overrepresented in the (+)-training set used in this analysis (Table 1; see also Methods). For instance, no overrepresentation has been found for important factors such as NF-κB, AP-1 and C/EBP. Nevertheless, these factors were included in the model, because not the binding sites themselves, but their combinations may be overrepresented.

**Table 1:** The genes of the (+)-training set (without orthologs). Marked with asterisks are those included in the "seed" set.

No	Gene name	Accessin no. And LocusLinkID	Experimental evidence	Additional information	Participation in anti-Pseudomonas response
1	Monocyte chemoattractant protein-1, MCP-1*	EMBL: **D26087**	Microarray [66], other experiments [20,21,38]	Is well know as expressed in antibacterial response	100%
2	β-defensin*	LocusLinkID: 1673	[15,18,19,39,40]	Is well known as expressed in antibacterial response; important target gene in innate immunity	100%
3	Interferon regulatory factor 1, IRF-1*	LocusLinkID: 3659	Microarray [66]	Known to be expressed in epithelial cells	probable
4	Equilibrate nucleoside transporter 1, SLC29a1	LocusLinkID: 2030	Microarray [66]		
5	Proteinkinase C η type, PKCη*	LocusLinkID: 5583	Microarray [66] TRANSPATH^®^	Important link in Ca^2+^-connected pathways	probable
6	Folypolyglutamate synthase, FPGS	Ensembl : **ENSG00000136877**	Microarray [66]		
7	RhoB*	LocusLinkID: 388	Microarray [66]	is induced as part of the immediate early response in different systems	probable
8	Origin recognition complex subunit 2, hORC2L	LocusLinkID: 4999	Microarray [66]		
9	Transcription factor TEL2*	LocusLinkID: 51513	Microarray [66]	Transcription factor	probable
10	Interleukin 8, IL8*	EPD: **EP73083**LocusLinkID: 3576	[10,11,26,44,45]	Is well know as expressed in antibacterial response	100%
11	Transcription factor ELF3*	LocusLinkID: 1999	Microarray [66]	Transcription factor	probable
12	Mucin 1(mouse gene), MUC1*	RefSeq: **NM_013605**	[17,27,28,36,47]	Different mucins are shown as expressed in antibacterial response	100%
13	NF-kappaB inhibitor alpha, IkBa*	LocusLinkID: 4792 EPD: **EP73215**	Microarray [66]	NF-kB inhibitor, the main link in NF-kB-targeting pathways	Very high
14	Tissue Factor Pathway Inhibitor 2, TFPI	LocusLinkID: 7980 EPD: **EP73430**	Microarray [66]		
15	Urokinase-type plasminogen activator precursor, PLAU	LocusLinkID: 5328	Microarray [66]		
16	c-jun*		Microarray [66]	Transcription factor	probable
17	Cytochrom P450 dioxin-inducible*	LocusLinkID: 1545	Microarray [66]	Stress-inducible	probable
18	Dyphtheria toxin resistance protein, DPH2L2	EPD: **EP74285**	Microarray [66]		

On the other hand, some of the factors, which have also been mentioned in literature as potentially relevant (e.g., SRF [30]) or might be of a certain interest because of their participation in relevant pathways (CREB, according to the TRANSPATH database [31]) were not included in the model because we could not adjust the thresholds for their detection according to our requirements (see Methods). SRF were of special interest, because it is known that it tends to cooperate with Elk-1 [30], but to identify 80% of TP we had to lower the matrix similarity threshold to 0.65, which is unacceptably low and would provide too many false positives.

Finally, we constructed our promoter model of binding sites of 5 TFs (NF-κB, C/EBP, AP-1, Elk-1, Sp1), considering their pairwise combinations and some combinations of higher order (complementary pairs, see below).

In several steps of the model construction we had to estimate overrepresentation of a feature in the (+)-training set compared with the (-)-training set. We operated with the number of sequences that possess the considered feature, in our case a pair of TFBS, at least once. Otherwise, mere enrichment of a feature in the (+)-training set may be due to strong clustering in a few members of that set which would not lead to a useful prediction model. At the first step the T-test has been performed (the normality of distribution has been demonstrated before (data no shown)), but it appeared to be a weak filter: for example, we could find several pairs which showed, if estimated with T-test, a remarkable overrepresentation (p < 0.001), but with a difference of 97% in the (+)-training set versus 85% in the (-)-training set, which is of no practical use to construct a *predictive *model, since it is also important to have minimal occurrence of a discriminating feature in the (-)-training set. In the further work we considered all pairs with p < 0.005, but as this did not reasonably restrict the list of considered pairs, we had to apply an additional filtering approach. For this purpose we used a simple characteristic such as the percentage of sequences in (+)- and (-)-training sets. By operating directly with percentages we could easily filter out those pairs which would identify too many false positive sequences, thus getting rid of a substantial part of useless information. This procedure allows to estimate immediately the applicability of the model to identify further candidate genes that may be involved in the cellular response under consideration (see *Methods*).

The main problem of promoter model construction are the numerous false positives. Developing our approaches we applied some anti-false-positives measures :

• distance assumptions

• identification of "seed" sequences

• phylogenetic conservation

• subclassification into complementary sequence sets.

In the following, we will comment on each item in more details.

### Distance assumptions

The commonly accepted view that functionally cooperating transcription factors may physically interact with each other triggered us to introduce certain assumptions concerning the distances between the considered TFBS. Transcription factors can interact either immediately with each other or through some (often conjectural) mediator proteins (co-factors). Principally there can be many ways of taking this into account, since our knowledge about the mechanisms of interaction is limited. In this work we used two different approaches to consider distances in the promoter model development.

In the first case we based our assumptions on the structure of known composite elements. We assumed that the binding sites of interacting TFs should occur in a distance of not more than 150 bp to each other (which is the case for most of the reported composite elements [17]; 150 bp is even an intended overestimation). To be on the safe side and not to overlook some potentially interesting interactions we allowed the upper threshold of 250 bp. Also by analogy with composite elements, for which it is relevant that the pair occurs not *at *a certain distance, but *within *a certain distance range, we considered the pairs occurring in segments of a certain length.

The second approach was based on more abstract considerations. Thinking of TF interaction, we can imagine three different situations:

(a) Directly interacting factors should have the binding sites at a close distance.

(b) The factors interacting through some co-factor may have binding sites on some medium distance, depending on the size and other properties of the co-factor (and the factors themselves).

(c) We can also expect direct interaction of another type, when the two factors are not located in the nearest neighborhood, but their interaction requires the DNA to bend or even to loop. This means that the distance is no longer a close one, although we cannot estimate the distance range for this case; thus, we allowed different ranges of distances, excluding only the closest ones.

We searched for pairs in three distance ranges, roughly called "close", "middle" and "far", all with adjustable borders, so that moving them we could get the best proportion of percentages in (+)- and (-)-training sets. We used the search in the distance ranges as a starting point, but some of the found pairs required optimization of the borders, so that they finally did not fit into any of the predefined ranges. The initial "close" range was taken as 5–20 bp, to exclude the overlapping of the sites, but to allow close interaction; however, the border had to be shifted in many cases up to 50 bp. The initial "middle" range was chosen from 21 to 140 bp (the number of nucleotides wrapping around the core particle of the nucleosome); the "long" range had its upper border at 250 bp.

### "Seed" sequences

Initially the idea of "seed" sequences was exploited because of the desire to make use of preexisting biological knowledge about the expressed genes and also because of doubts in the reliability of the available data set. Different experimental approaches differ in their reliability. The microarray analysis is not absolutely reliable [31,34-36], so we could expect that not all of the reported genes may be relevant for the antibacterial response. On the other hand, some genes are already known to be relevant according to additional published evidence. We thus decided to search for distinguishing features first in these "trustable" genes, and then to spread the obtained results to the whole set.

Therefore, we started our analysis with a group of "seed" sequences, which we considered for distinct reasons more reliable and preferable. Choosing a seed group, we took into consideration two kinds of evidence; the first was the source of information, i. e. the methods with which the gene has been shown to participate in the response. We took the promoter sequences of those genes which have been reported by other methods but microarray analysis [11,13,15,18-22,27-29,38-47,47], and which have been independently reported by at least two different groups.

The second kind of evidence was whether we could find any additional biological reasoning for the gene to participate in this kind of reply. For instance, a well-known participant of the NF-κB-activating pathway such as IκBα, or participants of different pathways which are likely to be triggered here as well, like c-Jun or PKC, were estimated as the first candidates for the "seed" group.

**Figure 1:**
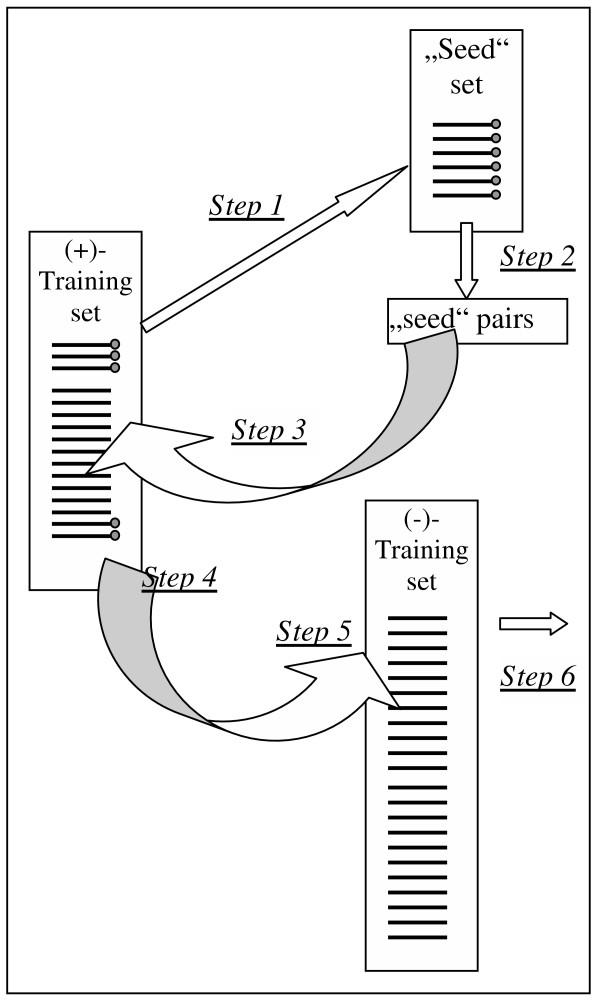
**Algorithm of the search for common pairs using seed sets. **Step 1. Selection of a "seed" set. Step 2. Identification of all pairs in the "seed" set; only those, which are found in 100% of the "seed" sequences, are taken into further consideration. Step 3. Search for the selected pairs in the whole (+)-training set. Step 4. Only those which are found in more than 80% of sequences of the (+)-training set are taken for into the further consideration. Step 5. Search for the "survived" pairs in the negative training set. Only those which are present in less than 40% of sequences are left. Step 6. The list of the common pairs is ready for the next analysis.

Finally, the "seed" contained 12 human sequences (Table 1). We could retrieve all mouse orthologs constituting a separate mouse "seed". We then run our analysis in either "seed" separately and in the combined human/mouse "seed" and compared the results. First, we identified all TFBS pairs that are present in all sequences of this "seed" group (see *Methods*) (Fig. 1, step 2). Further on, we searched for the found pairs in the whole (+)-training set (Fig. 1, step 3). In the next step we made a search in the (-)-training set for those pairs that were found in at least 80% of the (+)-training set (Fig. 1, step 4), choosing only those which showed the lowest percentages in the (-)-training set (Fig. 1, step 6).

Using this approach, we could avoid being drowned by a flood of pairs, most of which would be of minor importance. The huge number of nearly 37,000 pairs in different intervals which can be found in the whole (+)-training set was reduced by at least two orders of magnitude: depending on the "seed" the number of considered pairs varied from 50 to 400. In the next steps this number was reduced by another order of magnitude (Table 2).

**Table 2:** Stepwise filtering of pairs.

Pairs found on different steps of the search	No of found pairs
Pairs found in the whole training set in all distance intervals	~37000
Pairs found in the "seed" set in all distance intervals (step 2 on the fig. 1)	~400
"Seed" pairs in more than 80% of the training set (step 4 on the fig. 1)	~180
"Seed" pairs in more than 80% of the training set and less than 40% of the negative training set (step 6 on the fig. 1)	4

Each "seed" is characterized by its own set of pairs. To ensure the robustness of the obtained results, we undertook the "leave-one-out" test, removing consecutively one sequence of the "seed" set (for the combined "seed" sets which included human and mouse orthologs we excluded simultaneously both orthologous sequences). This has been repeated for each sequence (or ortholog pair). Only the robust pairs have been taken into further consideration.

### Phylogenetic conservation

Evolutionary conservation of a (potential) TFBS is generally accepted as an additional criterion for a predicted site to be functional (phylogenetic footprinting; [49-52]). However, some recent analysis of the human genome reported by Levy and Hannenhalli [50,53] and our own observations made for short promoter regions have shown that only about 50% [50], 64 % [53] or 70 % (Sauer et al., in preparation) of the experimentally proven binding sites are conserved. Missing between 30 and 50 % of all true positives may seem to be acceptable when analyzing single TFBS, but if one constituent of a relevant combination of TFBS belongs to a non-conserved region, we will loose the whole combination from all further analyses.

The observed fact is that functional features are not necessarily bound to conserved regions, as long as we speak about primary sequence conservation. Dealing with such degenerate objects as TF binding sites, one should not expect an absolute conservation of their binding sequences. From the functional point of view, it seems to be more reasonable to expect that not the sequences, but the mere occurrence of binding sites and/or their combinations as well as (perhaps) their spatial arrangement would be preserved among evolutionarily related genomes. That is the approach that we use in the present work, completely refraining from sequence alignments. We search for those pairs of TFBS which can be found in human and corresponding mouse orthologous promoter regions, considering the promoter as a metastring of TFBS. We took a feature (the pair of TFBS) into account only if we could identify it in both orthologous promoters, not taking into consideration in what region of the promoter it appeared; we also did not try to align metastrings of TFBS symbols, since they may be interrupted by many additional predicted TFBS (no matter whether they are true or false positives). While this work was in progress, we found a very similar approach in the work of Eisen and coworkers [54,55], who searched for conserved "word templates" in the transcription control regions of yeast. We believe that switching from primary sequence preservation to the conservation of higher-order features like clusters of TFBS is the next step in development of the approaches of comparative genomics.

### Complementary pairs (pairs of pairs)

**Figure 2:**
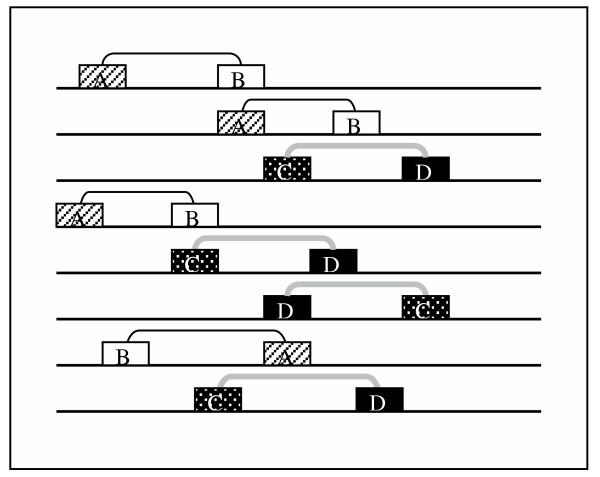
**Complementary pairs **A, B, C and D are transcription factor binding sites, which form two sorts of pairs (A-B and C-D). These pairs are complementary in the sense of occurring in complementary subsets of the whole set.

The idea that combinations or clusters of regulatory sites in upstream regions provide specific transcriptional control is not new [1,8,56]. Nevertheless, the problem of detecting such combinations is still under active development. As mentioned before, due to the complexity of the regulatory mechanisms in eukaryotes the computational prediction of functional regulatory sites remains a difficult task, and the spatial organization of the sites is the problem of the next level of complexity. To facilitate the search for combinations we tried to exploit the concept that subsets of principally co-regulated promoters may be subject to differential regulation. If the response of the cell is mediated through at least two distinct pathways, it is logical to suppose that there are subsets of promoters activated by each of them. The subsets may not be obvious from the expression data or from any other observations, but in some cases (as in ours, when we have two different pathways triggering the same response) one can presuppose the existence of two or more subsets, each of them possessing an own combination of TFBS. These combinations will be complementary in the sense of their occurrence in the set (Fig. 2). For simplicity we considered only pairs of TFBS, but the search for combinations of higher order would make the model more specific. Moreover, detection of complementary pairs enables to identify corresponding complementary subsets of sequences, thus to shed light on some features of the ascending regulatory network.

### Formalization of the approach

In the following, we will formalize our approach and describe the logics of our investigation.

All procedures are described for the example of pairwise combinations, but principally all of them can be applied to combinations of higher orders. We restricted our attempt to pairs for sake of computational feasibility.

### Identification of pairs

We consider all possible pairwise combinations of TFBS in each sequence, as described in *Methods*. A pair is taken into account if it has been found in a sequence at least once.

Let us consider two TFBS *m *and *n *located in a distance range from *r*_1 _to *r*_2 _(where *r*_1 _≤ *r*_2_) on either strand of DNA (+ or -). We can denote the sets of sequences containing pairs in different relative orientation as, .

**Figure 3:**
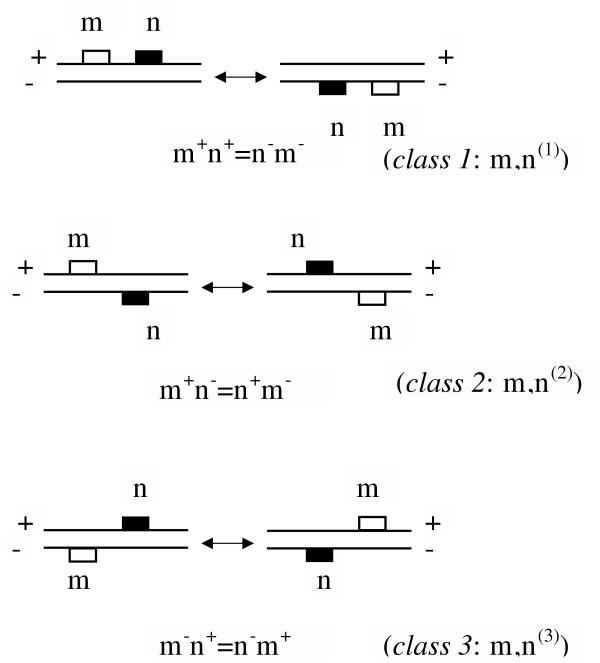
**Pair classes **When grouping different combinations of transcription factor binding sites according to mutual orientation, we allow inversions of the whole module. This gives rise to a total of three classes as shown.



To allow inversions of DNA segments containing pairs, we consider three classes of combinations (Fig. 3):

In more general form for *i *= 1,...3  represents the set of sequences with a pair of *i*-th class *m*, *n*^(*i*) ^(*r*_1_, *r*_2_).

Let  be a fraction of the sequences  in the (+)-training set, and  the fraction of sequences  in the (-)-training (control) set.

We have to solve now the optimization problem to maximize the difference  by choosing appropriate values for *m*, *n*, *i *and *r*_1_, *r*_2_. Also, we are interested only in pairs, which are present in at least a minimum fraction of (+)training sequences (*C*_1_) and in a defined maximum fraction of (-)-training sequences (*C*_2_). They can be filtered in advance.

Thus, we search for such  for which



where 0 ≤ *C*_1,2 _≤ 1 are adjustable parameters.

For single pairs we chose *C*_1 _= 0.8 and *C*_2 _= 0.4. We could not find pairs which would satisfy more stringent parameters, i. e. either higher *C*_1 _or lower *C*_2_; on the other hand, requirement (1) was found to be satisfied by a lot of different combinations which gave rise to the same *P*_*t *_and *P*_*c*_.

To make the analysis more specific, we can consider combinations of pairs instead of single pairs. For sake of simplicity, we will omit furtheron (*r*_1_, *r*_2_) from the expression  (but it should be kept in mind that  is always a function of (*r*_1_, *r*_2_)). Each possible type of pair is determined by values of *m*, *n *and *i*. We can list all types of pairs and assign a number *j *to each pair in this list. Then each type of pair is characterized by *m*_*j*_, *n*_*j*_, *i*_*j*_:



Then the sequences with the pair can be represented as . For simplicity, let us call



For two different *j*_1 _and *j*_2 _(*j*_1 _≠ *j*_2_) we can identify  and , which appear in the (+)training set simultaneously:



A triple or a combination of a higher order can be represented in the same way.

### Defining complementary pairs (pairs of pairs)

The antibacterial response of the cell is triggered by at least two distinct pathways, and it may be therefore supposed that there are subsets of promoters activated by each of them. Optimally, they should be "complementary" in the sense of appearing in complementary subsets of the (+)-training set (Fig 2).

**Figure 4:**
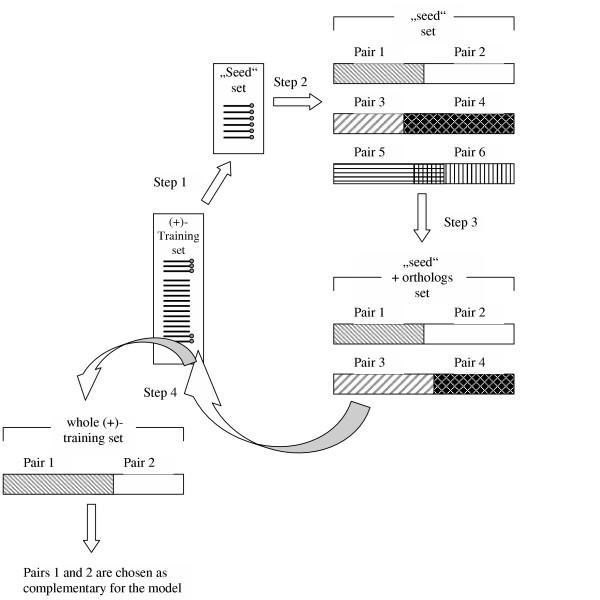
**Algorithm of the search for complementary pairs using "seed" sets **Step 1. Selection of a "seed" set; Step 2. Selection of complementary pairs in the human "seed"; every combination is checked in the (-) training set and only those, which are found in less than 40% of sequences, are taken into further consideration. Step 3. Selection of complementary pairs in the "seed" of orthologs or in the joint "human + orthologs" "seed". (Step 2 may be omitted and substituted by Step 3) Step 4. Search for the selected pairs in the whole (+)-training set. After that the final choice is made.

Complementary pairs were searched first in a "seed" subset of the (+)-training set of sequences (Fig 4, step 1). It comprises those 12 human genes for which the most reliable evidence is available that they are involved in the antibacterial response (as discussed in the subsection *Seed sequences*; Table 1). We considered all possible pairs which could be found in this subset (Fig. 4, step 2). Further on, we considered all pairwise combinations, calling pairs complementary, if:

(a) they together cover the whole subset (*C*_1 _is therefore always set to 1, );

(b) each of them can be found in not more and not less than a certain number of sequences (defined by adjustable parameters *C*_3 _and *C*_4_, see below), with an allowed overlap (defined by the parameter *C*_5_).

Thus, the requirement for complementary pairs is:



where 0 ≤ *C*_3,4,5 _≤ 1 are adjustable parameters.

We chose *C*_3 _= 0.3, *C*_4 _= 0.7 and *C*_5 _= 0.2. As we had no means to estimate the expected proportion of complementary pairs in the subsets, we started with these rather unrestrictive parameter settings. Finally the chosen pairs were found in the proportion 0.4/0.6 for *C*_3_/*C*_4_. In the next step we repeated the search including the orthologous sequences to the "seed" set (Fig. 4, step 3). We looked for those pair combinations which were found in the first step (in the human "seed" sequences). (The second and the third steps may be combined in one).

In the last step we repeated the search in the whole (+)-training set of 33 sequences, looking only for the combinations found in the second step (i.e., in the 12 "seed" and their orthologous sequences) (Fig. 4, step 4).

The percentage of the pair occurrence in the (-)-training set has been counted on the first step with the subsequent filtering of pairs.

### Results of the pair search

A rather large number of combinations satisfied the requirements described in the previous section. However, when we selected those that were robust in a "leave-one-out" test for the "seed" sets, the final list of potential model constituents was shortened down to only 2 ubiquitous and 12 complementary pairs.

We found one satisfactory pair which should be found in all promoters of target genes:

*AP *- 1, *NF *- *κB*^(1)^(10,93)

(*AP-1*, *NF *- *κB*, class 1, distance from 10 to 93 bp; see Fig. 3 for pair classes).

The search for the combination of two or more pairs, which should be found in the whole set simultaneously, did not give any significant improvement of the results.

**Figure 5:**
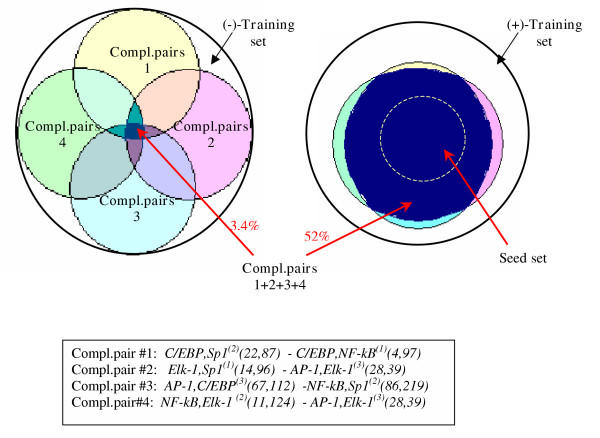
**Seven pairs, which are combined in four complementary combinations, and the results of their simultaneous application **Each of the complementary pairs searches for nearly the same portion of the training set, while in the negative training set their intersection appears to be very small. Here, only those pairs are shown that have been chosen for the final model, but there were several more, which searched for the same subset of the training set and gave altogether 1,7% in the negative training set. Note that the circles are not exactly drawn to scale.

Among the complementary pairs we found, several of them appeared to be interchangeable: each pair of pairs or any combination of them resulted in the selection of the same subsets from the (+)-training set (52%) (Fig. 5). Fig. 5 shows only those pairs which have been chosen for the final model, but there were several more which identified the same subset of the (+)-training set. The large number of complementary pairs may indicate that they are parts of more complex TFBS combinations, consisting of 4, 5 or more TFBS.

The false positive rate depended on the number of applied pairs; when we used all of them together, they gave only 1.7% of FP (i. e., only 1.7% of the sequences in the (-)-training set revealed the presence of all pairs under consideration). But the simultaneous usage of all the pairs could overfit the model, so we did not apply them all, sacrificing a bit of specificity for sake of a higher sensitivity.

Finally, we came up with 4 complementary pairs (Fig. 5) composed of 7 different TFBS pairs. Four of these TFBS pairs together are indicative for one subset of sequences, the remaining three for the other. As it has been mentioned before, the discovery of complementary pairs entails automatically the discovery of the corresponding subsets of sequences. We analyzed the distribution of the constituents of the found complementary pairs across the (+)-training set, which enabled us to assign the genes either to one or to the other subset, or to both (Table 3). Note that one of the subsets (subset 1) is in good agreement with the experimental data: MCP1, IL-8, β-defensin and MUC1 are known to be regulated by LPS, whereas IκBa is an important participant of this pathway; thus, these genes could be expected to belong to one pathway and, therefore, to one subset. Here, they all belong to the subset 1. This observation provides good support for the concept of complementary pairs which we applied here.

**Table 3:** Assignment of training sequences to two subsets. Genes marked with asterisk are known to be activated through LPS-dependent pathway; note that they all belong to one subset.

	Subset 1(LPS-dependent pathway)	Subset 2
Complementary pairs	Elk-1, NF-κB^(2) ^(11–124) Elk-1, Sp1^(1) ^(14–96) C/EBP, Sp1^(2) ^(22–87) C/EBP, NF-κB^(1) ^(4–97)	AP-1, Elk-1^(3) ^(28–39) NF-κB, Sp1^(2) ^(86–219)
Regulated genes (in the training set)	MCP1* IL8* β-Defensin* MUC1* ELF3 cytochrome p450 IkBa*	PKC, proteinkinase C TEL2 c-jun(?) TFPI-2
	RhoB, PLAU, IRF-1, hORC2L	
Not assigned	SLC29, DPH2L2, FPGS,	

In order to avoid the overfitting of the model and to demonstrate the significance of our results, we performed a permutation test. For that, we conducted 2000 iterations of random permutation of (+) and (-) labels in the training sets and tried to rebuild the model using the procedure described above. The rate of correct classification on this random selection was estimated. The cases of common and complementary pairs were considered separately. The analysis was made for different *C*_1_, *C*_2 _(0.7<*C*_1_<0.8, 0.4<*C*_2_<0.5) for common pairs; for complementary pairs we considered the case with *C*_3 _= 0.3 *C*_4 _= 0.7 *C*_5 _= 0.2. The probability to find by chance a "seed" of 12 sequences which would produce at least one pair common for the random selection of 33 sequences (including the "seed") depends on the chosen *C*_1_, *C*_2 _and is found to vary between p < 0.0005 (*C*_1 _= 0.*8*, *C*_2 _= 0.4, the parameters used for our model construction) and p = 0.02 (*C*_1 _= 0.7, *C*_2 _= 0.4). We failed to find any complementary pairs after 1000 iterations of the permutation test with the parameters used for the "real" (not permuted) model construction. These results suggest that the success of the model construction based on the search for combinations of TFBS is strictly dependent on the selected training set (thus, on our prior biological knowledge) and that the significance of the findings, depending on the correct choice of the adjustable parameters, is high enough to claim their non-randomness. Thus, we can say that in the described case the pairs found in the given (+)-training set with the given parameters are the real characteristics of this set.

### Promoter model

The model consists of two kinds of combinations of pairs: ubiquitous pairs (which should be found in all promoters of the target genes), and complementary pairs. We can divide the model into two modules, one for each kind of combination.

Let *M*1 and *M*2 be modules comprising ubiquitous pairs and complementary pairs, respectively.

Module *M1 *comprises the pair *AP*-1, *NF-κB*^(1)^(10,93).

Module *M2 *comprises all complementary combinations listed in the Fig. 5. Each complementary pair can be taken as a submodule (*m*) in *M2*.

To apply the model means to search for sequences containing all these combinations. Let us call *S(M) *the set of sequences which possess the whole model *M*; then we can also consider *S(M1) *and *S(M2) *(the sets possesing the modules *M1 *and *M2*, respectively), and *S(m) *– the set with a submodule *m*.

Then



Module M2 consists of submodules (*m*); in this case we consider four submodules, so the sequences containing M2 can be found as:

*S*(*M*2) = *S*(*m*_1_) ∩ *S*(*m*_2_) ∩ *S*(*m*_3_) ∩ *S*(*m*_4_),

where the set with each submodule we must consider as a union of sequence sets containing the complementary pairs:



The final result of application of the model M can be presented as

*S*(*M*) = *S*(*M*1) ∩ *S*(*M*2)

The model gives 3.4% of false positives and re-identifies 52% of the whole (+)-training set, but these 52% comprise all most reliable sequences of the set (remember that we must allow for some reduction because the set is not absolutely reliable).

### Identification of potential target genes

Applying our promoter model to screening of 13000 upstream regions from a collection of human 5'-flanking sequences [57], we identified about 580 genes as harboring this combination of TFBS. After erasing all those that encode hypothetical products, we came up with a list of 430 potential target genes, which can be checked for plausibility. More than 60% of these genes encode different representatives of the immune system, which can be expected to participate in the cells' response, as well as transcription factors and other regulatory proteins. Some of the most interesting potential target genes are shown on the Table 4. The whole data set one can find in the Additional files.

**Table 4:** Selection of candidate genes identified by the promoter model. The whole list one can find in Additional files.

TNFRSF14 tumor necrosis factor receptor superfamily TNFAIP6 tumor necrosis factor, alpha-induced protein 6 PPP3CA protein phosphatase 3 (calcineurin A) NLI-IF nuclear LIM interactor-interacting factor WISP1 WNT1 inducible signaling pathway protein 1 IL8 interleukin 8 TFPI2 tissue factor pathway inhibitor 2 DEFB2 defensin, beta 2 POU2F1 POU domain, class 2, transcription factor 1 MAP2K1IP1 mitogen-activated protein kinase kinase 1 interacting protein 1 CSF2 colony stimulating factor 2 (granulocyte-macrophage) TAF2F TATA box binding protein (TBP)-associated factor RNA polymerase II, F, 55 kD ABT1 TATA-binding protein-binding protein CALN1 calneuron 1 TRAF1 TNF receptor-associated factor 1 FPGS folylpolyglutamate synthase RENT2 regulator of nonsense transcripts 2 CYP26A1 cytochrome P450, subfamily XXVIA EHF ets homologous factor, MAP3K11 mitogen-activated prot. kinase kinase kinase 11 IRAK-M interleukin-1 receptor-associated kinase M ARHGDIA Rho GDP dissociation inhibitor (GDI) alpha HSY11339 GalNAc alpha-2, 6-sialyltransferase I, long form	HCNGP transcriptional regulator protein CYP4F11 cytochrome P450, subfamily IVF IRF3 interferonregulatory factor 3 ICAM3 intercellular adhesion molecule 3 PPARA peroxisome proliferative activated receptor, alpha IKBKG inhibitor of kappa light polypeptide gene enhancer in B-cells, kinase gamma ELK1 ELK1, member of ETS oncogene family STK31 serine/threonine kinase 31 SERPING1 serine (or cysteine) proteinase inhibitor GPR4 G protein-coupled receptor 4 RAB5B RAB5B, member RAS oncogene family RAB7 RAB7, member RAS oncogene family NFKB1 nuclear factor of kappa light polypeptide gene enhancer in B-cells NFKBIB nuclear factor of kappa light polypeptide gene enhancer in B-cells inhibitor, beta CEBPE CCAAT/enhancer binding protein (C/EBP), ε ELK1 ELK1, member of ETS oncogene family EHF ets homologous factor *15 Zinc finger proteins* *small inducible cytokine subfamily A (Cys-Cys), members 5,11, 20 and 23* *Interleukins: *IL1, IL1delta, IL8, IL12A, IL12B, IL13, IL23,

## Discussion

We have proposed some approaches to promoter model construction and show how these approaches work in the particular case of antibacterial response of a eukaryotic cell, namely the reaction of human lung epithelial cell to *P. aeruginosa *binding. One of the results of our work is a list of potential target genes, enriched with different regulatory proteins, including transcription factors and known participants of the ascending pathways. This theoretical result must have two practical consequences: first, it allows to restrict further experimental research to a manageable number of candidate genes; second, it enables to understand or to clarify some uncertain details concerning the triggering pathways, and thus to make some new predictions based on this information. There is a number of published tools for searching for regulatory modules (i.e., "sequence elements that modulate transcription", following the definition given by Bailey and Noble [1] following [7,58]) [7,53,59-63]. The used algorithms may be devided in three classes (sliding window approach, hidden Markov models, discriminative technique), as briefly reviewed in [1]. Any of the approaches, independent of which algorithm it is based on, encounters the same problems arising from the biological nature (and extreme complexity) of the object: (i) scarcity of knowledge about exact location of promoters and enhancers and of experimentally proven binding sites (information used for constructing (+)-training sets); (ii) the fact that statistical significance of a feature (TFBS or a cluster of them) does not necessarily tell anything about the biological functionality of this feature; analogously, the insignificance can not be taken as a proof of the lack of function; (iii) usually weak reasoning for grouping genes (their promoters) in sets according to their function, co-regulation, functional occurrence in the same cell types, etc. The latter has some lucky exceptions, like sets of muscle genes [58] or cell-cycle regulated genes [64], and the situation will obviously improve with further development of microarray technique.

In the present work we tried to address the listed problems. We could not, of course, improve the situation with the paucity of experimental data, only endeavored to make our data searches as accurate and exhaustive as possible. In principle we developed our approaches basing them, whenever possible, on biological reasoning. We find it extremely important to use as much experimental evidence as it is available at the moment. In our approach we alternated two different kinds of steps – expanding the data and restricting it: exhaustive data search – "seed" and distance constraints – exhaustive enumeration of all possible pairs – complementary pair constraints.

To avoid the problem of low confidence in the (+)-training set (which may occur not only in our specific case), we developed the approach of "seed" sequences. The difference from the "seeds" used in cluster analysis is that in our approach the choice of the "seed" is biologically based. Although the "seed" approach is, obviously, a restrictive measure, moreover, a pre-process restriction, which may result in missing potentially relevant additional sequence features, we find it useful and appropriate when the choice of the "seed" is made on a solid biological basis. After having applied the restrictive "seed" technique and distance assumptions, we undertake an exhaustive, complete enumeration of all possible pairs of potential TF binding sites that can be found in the (+)-training set, which in turn reveals a large number of combinations. This list of all found pairs is processed under a new kind of constraints imposed by the search for complementary pairs.

The search for complementary pairs is a completely new approach, which supplies us with a new kind of information. It enables to identify subsets of the (+)-training set which possess different regulatory modules, thus suggesting their triggering by different regulatory pathways. This kind of information becomes extremely important in two cases: (i) when two or more pathways are presupposed to be triggered in the cellular response, like in the case considered in this work; (ii) when the (+)-training set consists of not really co-regulated, but of co-expressed genes, without precise information about which of them are regulated by the same mechanism. The identification of complementary pairs and, consequently, groups of sequences enables to better define the co-regulated genes thus providing a partial, although only predicted, confirmation of the co-regulation, and at the same time to better understand the ascending pathways.

The final result of our search supported the idea of complementary pairs. There is a lot of evidence in literature that interleukin 8, β-defensin, monocyte chemoattractant protein and different mucins are regulated through LPS-triggered pathway(s) [12,15,38]. On the other hand, it is also well-known that LPS is one of the "gates" through which the antibacterial response is triggered [24,65]. We know, that in the particular case of interaction with *P. aeruginosa *this pathway is not the only one [13], but we do not know in advance which of the genes in the (+)-training set belongs to which pathway (except for several genes as listed above). We had no means to include our pre-knowledge in the search. With the complementary pair approach we could re-identify the LPS subset in good agreement with our expectations (Table 3), confirming the efficiency of the method.

Our approach, as any other, has its limits. It has been shown for the genuine composite elements of certain types (for instance, NF-AT and AP-1) [66] that one of the two constituents of a composite element could be rather degenerate, as compared with its canonical consensus sequence or when scored with a positional weight matrices (PWM). This means, that our requirement for all binding sites to be found with rather high PWM thresholds may be too restrictive. We are running risk to overlook those constituents of pairs which possess weak consensi. We could not find a solution to this problem. We have no information about which of the TFs could be represented by such low-threshold consensus, and if we take from the very beginning the lower thresholds for all considered matrices, we will be drowned in potential binding sites, nearly all of them probably being false positives. Nevertheless, we find that the PWM approach is better than string identification, which even with allowed mismatches can not provide the same flexibility as PWMs.

The next source of limitations we see in the preselection of factors according to published data. Obviously, we can not expect that the experimental data is exhaustive; some of the transcription factors may be not reported just because their participance in a certain process has not yet been investigated. On the other hand, statistical overrepresentation, as it has already been mentioned before, can not be taken by itself as proof of biological functionality or its lack; some TFBS cannot be overrepresented due to their degenerate nature. We had no other idea of how to take into account those TFBS which are not overrepresented, but to rely on published experimental data. We find that the usual methods based on statistical overrepresentaion are even more restrictive, but maybe the best solution could be found in merging both approaches – i.e., using the experimental evidence along with statistical ones, for instance using Bayesian techniques.

We see the perspectives of this work in two different fields: further investigation of regulatory networks triggered by *P. aeruginosa *binding, and further development of the methodological approaches, making them more flexible and applicable to any similar task. The list of predicted target genes has to be evaluated experimentally, but may have its value for further research already on the present step. The future work on reconstructing the intracellular pathways triggering the genetic program of the antibacterial cell response will be well supported with the information picked up from this list. It may give some hints for the next steps of experimental research, for instance providing information about the first candidates to be checked. The information about the complementary subsets of regulated genes helps to better understand the triggering pathways, and the complementarity of their function is a subject for further consideration.

The methodological approaches presented in this paper can be, of course, applied to other objects. In this work we focused on the experimentally proven basis for the initial choice of transcription factors. This kind of evidence is stronger than any prediction, but it can work only when this information is available, which may be not the case for some other sets of genes or cellular situations. In the next step of development we would like to allow also an exhaustive computational search through the whole list of known TFs for potential constituents of the models. The usage of Bayesian techniques, as mentioned in the previous paragraph, would be also appropriate for this kind of predictions.

## Conclusions

We suggest a methodology for promoter model construction based on the search of TFBS pairs and show how it works in the particular case of antibacterial response of human lung epithelial cells. We show that the method allows to identify and predict subsets of target genes potentially triggered by different regulatory pathways and thus possessing different regulatory modules. The methodology is easily applicable to any similar task and does not depend on the number of included TFs and/or number of investigated sequences, which only should not be too low for statistical reasons.

## Methods

### Databases

Eukaryotic Promoter Database , release 77-1.

DBTSS, the database of transcription start sites , release 3.0

TRANSCompel^® ^Professional release 7.1 

TRANSFAC^® ^Professional release 7.1 

TRANSPATH^® ^Professional release 4.1 

### Training sets

The positive (+) training set comprises:

1. Promoters of human genes shown to be expressed in epithelial cells after interaction with *P. aeruginosa *by means of:

a. microarray analysis [67],

b. other methods [11,13,15,27,28,37,38]. (Table 1)

2. Orthologous mouse promoters.

The sequences were derived either from Eukaryotic Promoter Database , or from DBTSS, the database of proven transcription start sites . The length of the sequences was 600 bp (-500/+100). This region comprises most of then known upstream elements and corresponds to the upstream region used by Davuluri *et al*. as "proximal promoters" for promoter recognition [69], plus a 100 bp proximal downstream region which also contains many known regulatory elements documented in the TRANSFAC database [70].

The "seed" set is a subset of the positive training set selected for highest experimental reliability (see Table 1).

The negative (-) training set was composed of randomly chosen 5'-upstream sequences derived from the TRANSGENOME information resource of annotated human genome features [57]. The set was manually cleaned from all genes which potentially could be involved in the same or similar cellular responses. The set comprised 2040 sequences.

### Defining the set of transcription factors (potential constituents of the model)

We based our selection of TFs on experimental evidence. For that we undertook an extended literature search, looking for the TFs which have been shown to take part either directly in the response of epithelial cells to *P. aeruginosa *binding or in the pathways triggered during similar responses. The search revealed 5 candidate factors: NF-κB [11,12,15,18,21,23,24,26], C/EBP [21,24,25,27], AP-1 [24,25], Elk-1 [16,24] and Sp1 [28,29,48].

Including C/EBP and Sp1 in the list was additionally reasoned by the fact that these factors are known to be second constituents in the most frequent NF-κB-containing composite elements as they are compiled in the TRANSCompel^® ^database [17]. Moreover, these are the types of composite elements known to participate in different kinds of immune response.

### Search for the potential transcription factor binding sites

We made this search with the weight matrix approach using the Match™ tool [68]; the matrices were chosen from the library collected in TRANSFAC^® ^[70]. For the model construction, the thresholds for the matrix search have been defined individually for each matrix and in such a way that (i) it should yield not less than 80% TP (true positive set, here the set of experimentally proven TFBS from TRANSFAC^®^); (ii) at least one hit for every searched transcription factor could be found in every sequence of the (+)-training set. The lower border for the thresholds was predefined as 0.80/0.79 (core similarity/ matrix similarity).

### Identification of pairs

We considered all the coordinates (with strand information) of all potential TF binding sites found by Match™ for each transcription factor. Further on, we examined all possible combinations of the coordinates, thus revealing all possible pairs in the sequence.

We worked under two different kinds of distance assumptions as described in *Formalization of the approach*, choosing the most promising results achieved with either of them. We considered all pairs of TFs within these segments. All the pairs of one type found within one distance range were merged. We considered a pair only if it appeared in the sequence at least once (within a certain distance), not taking in account the number of pairs in each sequence.

## Authors' contributions

ES developed the methodological approaches as well as statistical analysis and conducted the data analysis. EW conceived the study and participated in its design and coordination. Both authors drafted the manuscript. Both authors read and approved the manuscript.

## Appendix 1

### Estimation of the validity of model construction algorithm

The question is, if we choose by chance a subset of sequences, will our algorithm be able to define a model, specific to such a random subset? In other words, will this algorithm allow to make a model of anything, without dependence on the preselection of the sets ((+)-training set and/or the "seed" set)? We tried to prove the validity of the algorithm theoretically.

Our algorithm is based on the definition of biologically relevant "seed" sets, in which we search for the candidate pairs (normal and complementary ones). Therefore, in order to answer the question, it is reasonable to estimate the probability to come across a "seed" set of *k *sequences, 100% of which possess the required common feature: a pair, a combination of pairs or complementary pairs, just by chance. Note that this estimation is written not for the whole model construction process, but only for the first step of it, where we consider only the "seed" sequences.

Let us consider the frequencies of predicted single sites (*f*) of the TFs included in the model and the frequencies of all possible pairs (*F*), constructed of these sites. If the frequencies of single sites and the pairs of them satisfy the equation

*F*_*ij *_= *f*_*i *_*f*_*j*_,     (1)

we can interpret *F*_*ij *_as the probabilities of independent events, which is a prerequisite for the following formalism.

We measured the frequencies of predicted single sites and the frequencies of all possible pairs in the (-)-training set (see *Methods*). We did not take into consideration distances and orientations; the probability estimated for the general case will decrease further with the addition of new constraints.

The frequencies *f*_*i *_and *F*_*ij *_of single sites and pairs, respectively, were measured directly as

*f*_*i *_= *m*_*i *_/ *N*

*F*_*ij *_= *M*_*ij *_/ *N*

where *N *is the number of all sequences of the (-)-training set, *m*_*i *_is the number of sequences possessing the *i*-th site, and *M*_*ij *_is the number of sequences possessing pairs of the *i*-th and *j*-th sites. *F*_*ij *_was then calculated as (1) and compared with the measured value.

For all cases investigated in this work, the difference between the calculated and measured values did not exceed standard deviation (σ), only in one case getting to 1,5 σ (data not shown). This confirms the correctness of using pair frequencies as probabilities in this case.

Let us estimate the probability *P*_*pair *_to find a set of *k *sequences in *N *with any (at least one) pair, same in all *k*. We can enumerate all possible pairs of sites of the considered TFs, considering only the cases of the independent sites (*i*<*j*). Let U be the number of all possible pairs, then we can call

*F*_*ij *_= *F*_*u*_,

*u *∈ {1,..., *U*}

It is easy to show, that the probability *P*_*pair *_can be calculated as:



Let us estimate the probability *P*_2*pairs *_to find *k *sequences with any common pairwise combination of pairs (pair of pairs). The pairs of pairs may consist either of 3 (when one site is shared) or of 4 different sites (thus leaving out combinations of identical pairs); their probabilities therefore are:



and



where *f*_*i*_*, f*_*j*_*, f*_*l*_*, f*_*o *_are the frequencies of the single sites of the considered TFs, *i*<*j*<*l*<*o*.

We can enumerate all possible pairs of pairs (notating them as Q):



Let *V *be the number of all possible pairs of pairs, *V *= *t *+ *s*.

Analogously to (2), the probability to find *k *sequences each possessing a pair of pairs of one type, is:



where *v *∈ {1,..., *V*}.

Let us estimate the probability to find *k *sequences with any complementary pair (complementary combinations). We consider pairs as complementary, if two of them are found in the seed set in not more than 60% of the sequences and not less than 40 %, the allowed overlap being 20%. The two complementary pairs together must cover the whole seed set. In the case studied here, comprising the 12 sequences of the seed set, we fixed that each of the pairs should be present in at least 5, but not more than 7 sequences, and they are allowed to co-occur in 0–2 sequences.

The probability that we choose 12 sequences, possessing any one pair of complementary pairs in accordance with these requirements can be calculated as:



where *u*, *w *∈ {1,..., *U*}, and  are the binomial coefficients (note that this formula implies that *P*_*compl *_reaches the maximum when the frequencies of both pairs are 0.5).

All the probabilities were calculated for the (-)training set of 2040 5'-upstream sequences and for the set of 5 selected transcription factors (see *Methods*). The results are:

*P*_*pair *_= 0.44 ± 0.02

*P*_2*pairs *_= 0.13 ± 0.01

*P*_*compl *_= 0.013± 0.003

We have estimated the simplest variant, considering each time only one feature (1 pair, 2 pairs, or complementary pairs). In this case it can be seen that the simultaneous occurrence of 1 or 2 pairs in 12 randomly chosen sequences has a rather high probability, and thus we can not base our model construction on the search of only these features. (An increase of the number of "normal" pairs in the search will not dramatically improve the situation: the formula (3) describes the probability to find any combination of 3 or 4 sites, therefore, up to 6 pairs;obviously, the simultaneous search of more than 6 pairs will definitely overfit the model, so we do not consider this case). The probability to find 12 sequences sharing complementary pairs is much lower, so the consideration of a complementary combination makes the model much more specific, and the probability of finding a model with a complementary pair "by chance" is sufficiently low for us to claim that the proposed algorithm is valid. Note that this is a very rough estimation, considering only the upper borders; we would like to emphasize once more, that the probabilities were calculated without considering orientation and distance constraints, and that this is the estimation made for only the very first step of analysis: choosing of a seed set with needed properties. Obviously, this value depends on the number of the sequences in the "seed". Note that when we spread our requirements for simultaneous search on the whole (+)-training set (which is the next step of the model construction) the probability of constructing a model "by chance" will drop dramatically.

## Supplementary Material

Additional File 1 The whole list of genes found with the promoter model when applying it to the collection of 13000 human 5'-upstream sequences. This list is not cleaned from hypothetical genes. Click here for file

Additional File 2 The list of genes (found with the promoter model when applying it to the collection of 13000 human 5'-upstream sequences) cleaned from hypothetical genes. Click here for file

## References

[B1] Bailey TL, Noble WS (2003). Searching for statistically significant regulatory modules. Bioinformatics.

[B2] Brazma A, Jonassen I, Vilo J, Ukkonen E (1998). Predicting gene regulatory elements in silico on a genomic scale. Genome Res.

[B3] Fickett JW, Wasserman WW (2000). Discovery and modeling of transcriptional regulatory regions. Curr Opin Biotechnol.

[B4] van Helden J (2003). Regulatory sequence analysis tools. Nucleic Acids Res.

[B5] van Helden J, Andre B, Collado-Vides J (1998). Extracting regulatory sites from the upstream region of yeast genes by computational analysis of oligonucleotide frequencies. J Mol Biol.

[B6] Klingenhoff A, Frech K, Werner T (2002). Regulatory modules shared within gene classes as well as across gene classes can be detected by the same in silico approach. In Silico Biol.

[B7] Krivan W, Wasserman WW (2001). A predictive model for regulatory sequences directing liver-specific transcription. Genome Res.

[B8] Wagner A (1999). Genes regulated cooperatively by one or more transcription factors and their identification in whole eukaryotic genomes. Bioinformatics.

[B9] Werner T, Fessele S, Maier H, Nelson PJ (2003). Computer modeling of promoter organization as a tool to study transcriptional coregulation. FASEB J.

[B10] DiMango E, Ratner AJ, Bryan R, Tabibi S, Prince A (1998). Activation of NF-κB by adherent *Pseudomonas aeruginosa *in normal and cystic fibrosis respuratory epithelial cells. J Clin Invest.

[B11] Smith RS, Fedyk ER, Springer TA, Mukaida N, Iglewski BH, Phipps RP (2001). IL-8 production in human lung fibroblasts and epithelial cells activated by the *Pseudomonas aeruginosa *autoinducer N-3-oxodododecanoyl homoserine lactone is transcriptionally regulated by NF-κB and activator protein-2. J immunol.

[B12] Zhang G, Ghosh S (2001). Toll-like receptor-mediated NF-kB activation: a phylogenetically conserved paradigm in innate immunity. J Clin Invest.

[B13] McNamara N, Khong A, McKemy D, Caterina M, Boyer J, Julius D, Basbaum C (2001). ATP transduces signals from ASGM1, a glycolipid that functions as a bacterial receptor. Proc Natl Acad Sci USA.

[B14] Britigan BE, Railsback MA, Cox CD (1999). The *Pseudomonas aeruginosa *secretory product pyocyanin inactivates α_1 _protease inhibitor: implications for the pathogenesis of cystic fibrosis lung disease. Infect Immun.

[B15] Harder J, Meyer-Hoffert U, Teran LM, Schwichtenberg L, Basrtels J, Maune S, Schroeder J-M (2000). Mucoid *Pseudomonas aeruginosa*, TNFα, and IL-1β, but not IL-6, induce human β-defensin-2 in respiratory epithelia. Am J Respir Cell Mol Biol.

[B16] Guha M, O'Connell MA, Pawlinski R, Hollis A, McGovern P, Yan SF, Stern D, Mackman N (2001). Lipopolysaccharide activation of the MEK-ERK1/2 pathway in human monocytic cells mediates tissue factor and tumor necrosis factor alpha expression by inducing Elk-1 phosphorylation and Egr-1 expression. Blood.

[B17] Wingender E, Kel AE, Kel OV, Karas H, Heinemeyer T, Dietze P, Knueppel R, Romaschenko AG, Kolchanov NA (1997). TRANSFAC, TRRD and COMPEL: towards a frederated database system on transcriptional regulation. Nucleic Acids Res.

[B18] Li J-D, Feng W, Gallup M, Kim J-H, Kim J, Kim Y, Basbaum C (1998). Activation of NF-kB via a Src-dependent Ras-MAPKpp90rsk pathway is required for *Pseudomonas aeruginosa*-induced micin overproductionin epithelial cells. Proc Natl Acad Sci USA.

[B19] Diamond G, Kaiser V, Rhodes J, Russell JP, Bevins C (2000). Transcriptional regulation of b-defensin gene expression in tracheal epithelial cells. Infection and immunity.

[B20] Diamond G, Jones DE, Bevins CL (1993). Airway epithelial cells are the site of expression of a mammalian antimicrobial peptide gene. Proc Natl Acad Sci U S A.

[B21] Ko YH, Delannoy M, Pedersen PL (1997). Cystic fibrosis, lung infections, and a human tracheal antimicrobial peptide (hTAP). FEBS letters.

[B22] Ratner A, Bryan R, Weber A, Nguyen S, Barnes D, Pitt A, Gelber S, Cheung A, Prince A (2001). Cystic fibrosis pathogens activate Ca2+-dependent mitogen-activated protein kinase signaling pathways in airway epithelial cells. J Biol Chem.

[B23] Voynow JA, Young LR, Wang Y, Horger T, Rose MC, Fischer BM (1999). Neutrophil elestase increases MUC5AC mRNA and protein expression in respiratory epithelial cells. Am J Physiol.

[B24] Guha M, Mackman N (2001). LPS induction of gene expression in human monocytes. Cell Signal.

[B25] Ben-Baruch A, Michiel DF, Oppenheim JJ (1995). Signals and receptors involved in recruitment of inflammatory cells. J Biol Chem.

[B26] Bergmann M, Hart L, Lindsay M, Barnes PJ, Newton R (1998). IkappaBalpha degradation and nuclear factor-kappaB DNA binding are insufficient for interleukin-1beta and tumor necrosis factor-alpha-induced kappaB-dependent transcription Requirement for an additional activation pathway. J Biol Chem.

[B27] Leidal KG, Munson KL, Denning GM (2001). Small molecular weight secretory factors from Pseudomonas aeruginosa have opposite effects on IL-8 and RANTES expression by human airway epithelial cells. Am J Respir Cell Mol Biol.

[B28] Kovarik A, Lu PJ, Peat N, Morris J, Taylor-Papadimitriou J (1996). Two GC boxes (Sp1 sites) are involved in regulation of the activity of the epithelium-specific MUC1 promoter. J Biol Chem.

[B29] Perrais M, Pigny P, Ducourouble MP, Petitprez D, Porchet N, Aubert JP, Van Seuningen I (2001). Characterization of human mucin gene MUC4 promoter: importance of growth factors and proinflammatory cytokines for its regulation in pancreatic cancer cells. J Biol Chem.

[B30] Dieterich C, Herwig R, Vingron M (2003). Exploring potential target genes of signaling pathwas by predicting conserved transcription factor binding sites. Bioinformatics.

[B31] Krull M, Voss N, Choi V, Pistor S, Potapov A, Wingender E (2003). TRANSPATH^®^: an integrated database on signal transduction and a tool for array analysis. Nucleic Acids Res.

[B32] Pritchard CC, Hsu L, Delrow J, Nelson PS (2001). Project normal: defining normal variance in mouse gene expression. Proc Natl Acad Sci U S A.

[B33] Pan WA (2002). Comparative review of statistical methods for discovering differentially expressed genes in replicated microarray experiments. Bioinformatics.

[B34] Draghici S, Kulaeva O, Hoff B, Petrov A, Shams S, Tainsky MA (2003). Noise sampling method: an ANOVA approach allowing robust selection of differentially regulated genes measured by DNA microarrays. Bioinformatics.

[B35] Lee ML, Kuo FC, Whitmore GA, Sklar J (2000). Importance of replication in microarray gene expression studies: statistical methods and evidence from repetitive cDNA hybridizations. Proc Natl Acad Sci U S A.

[B36] Bilke S, Breslin T, Sogvardsson M (2003). Probabilistic estimation of microarray data reliability and underlying gene expression. BMC Bioinformatics.

[B37] Walsh DE, Greene CM, Carroll TP, Taggard CC, Gallagher PM, O'Neill SJ, McElvaney NG (2001). Interleukin-8 up-regulation by neutrophil elastase is mediated by MyD88/IRAK/TRAF-6 in human bronchial epithelium. J Biol Chem.

[B38] Becker MN, Diamond G, Verghese MW, Randell SH (2000). CD14-dependent lipopolysaccharide-induced b-defensin-2 expression in human tracheobronchial epithelium. J Biol Chem.

[B39] Sar B, Oishi K, Wada A, Hirayama T, Matsushima K, Nagatake T (2000). Induction of monocyte chemoattractant protein-1 (MCP-1) production by Pseudomonas nitrite reductase in human pulmonary type II epithelial-like cells. Microb Pathog.

[B40] Singh PK, Jia HP, Wiles K, Hesselberth J, Liu L, Conway BA, Greenberg EP, Valore EV, Welsh MJ, Ganz T, Tack BF, McCray PB (1998). Production of beta-defensins by human airway epithelia. Proc Natl Acad Sci U S A.

[B41] Liu L, Wang L, Jia HP, Zhao C, Heng HH, Schutte BC, McCray PB, Ganz T (1998). Structure and mapping of the human beta-defensin HBD-2 gene and its expression at sites of inflammation. Gene.

[B42] Zhao Z, Qian Y, Wald D, Xia YF, Geng JG, Li X (2003). IFN regulatory factor-1 is required for the up-regulation of the CD40-NF-kappa B activator 1 axis during airway inflammation. J Immunol.

[B43] Fritz G, Kaina B (2001). Transcriptional activation of the small GTPase rhoB by genotoxic stress is regulated via a CCAAT element. Nucleic Acids Res.

[B44] Gnad R, Kaina B, Fritz G (2001). Rho GTPases are involved in the regulation of NF-kB by genotoxic stress. Exp Cell Res.

[B45] Sar B, Oishi K, Matsushima K, Nagatake T (1999). Induction of interleukin 8 (IL-8) production by Pseudomonas nitrite reductase in human alveolar macrophages and epithelial cells. Microbiol Immunol.

[B46] Mori N, Oishi K, Sar B, Mukaida N, Nagatake T, Matsushima K, Yamamoto N (1999). Essential role of transcription factor nuclear factor-kappaB in regulation of interleukin-8 gene expression by nitrite reductase from Pseudomonas aeruginosa in respiratory epithelial cells. Infect Immun.

[B47] Sar B, Oishi K, Wada A, Hirayama T, Matsushima K, Nagatake T (1999). Nitrite reductase from Pseudomonas aeruginosa released by antimicrobial agents and complement induces interleukin-8 production in bronchial epithelial cells. Antimicrob Agents Chemother.

[B48] Gum JR, Hicks JW, Kim YS (1997). Identification and characterization of the *MUC2 *(human intestinal mucin) gene 5'-flanking region: promoter activity in cultured cells. Biochem J.

[B49] Duret L, Bucher P (1997). Searching for regulatory elements in human noncoding sequences. Curr Opin Struct Biol.

[B50] Levy S, Hannenhalli S, Workman C (2001). Enrichment of regulatory signals in conserved non-coding genomic sequence. Bioinformatics.

[B51] Hardison RC (2003). Comparative Genomics. PLoS Biol.

[B52] Pennacchio LA, Rubin EM (2003). Comparative genomic tools and databases: providing insights into the human genome. J Clin Invest.

[B53] Hannenhalli S, Levy S (2002). Predicting transcription factor synergism. Nucleic Acids Res.

[B54] Chiang DY, Moses AM, Kellis M, Lander ES, Eisen MB (2003). Phylogenetically and spatially conserved word pairs associated with gene-expression changes in yeasts. Genome Biol.

[B55] Moses AM, Chiang DY, Kellis M, Lander ES, Eisen MB (2003). Position specific variation in the rate of evolution in transcription factor binding sites. BMC Evol Biol.

[B56] GuhaThakurta D, Stormo GD (2001). Identifying target sites for cooperatively binding factors. Bioinformatics.

[B57] Kel-Margoulis OV, Tchekmenev D, Kel AE, Goessling E, Hornischer K, Lewicki-Potapov B, Wingender E (2003). Composition-sensitive analysis of the human genome for regulatory signals. In Silico Biol.

[B58] Wasserman WW, Fickett JW (1998). Identification of regulatory regions which confer muscle-specific gene expression. J Mol Biol.

[B59] Frech K, Danescu-Mayer J, Werner T (1997). A novel method to develop highly specific models for regulatory units detects a new LTR in GenBank which contains a functional promoter. J Mol Biol.

[B60] Kondrakhin YV, Kel A, Kolchanov NA, Romashchenko AG, Milanesi L (1995). Eukaryotic promoter recognition by binding sites for transcription factors. Comput Appl Biosci.

[B61] Prestridge D (1995). Predicting PolII promoter sequences using transcription factor binding sites. J Mol Biol.

[B62] Berman BP, Nibu Y, Pfeiffer BD, Tomanchak P, Celniker SE, Levine M, Rubin GM, Eisen MB (2002). Exploiting transcription factor binding site clustering to identify cis-regulatory modules involved in pattern formation in the Drosophila genome. Proc Natl Acad Sci U S A.

[B63] Markstein M, Markstein P, Markstein V, Levine MS (2002). Genome-wide analysis of clustered Dorsal binding sites identifies putative target genes in the Drosophila embryo. Proc Natl Acad Sci U S A.

[B64] Kel AE, Kel-Margoulis OV, Farnham PJ, Bartley SM, Wingender E, Zhang MQ (2001). Computer-assisted identification of cell cycle-related genes: new targets for E2F transcription factors. J Mol Biol.

[B65] Takeuchi O, Akira S (2001). Toll-like receptors; their physiological role and signal transduction system. Int Immunopharmacol.

[B66] Kel A, Kel-Margoulis O, Babenko V, Wingender E (1999). Recognition of NFATp/AP-1 composite elements within genes induced upon the activation of immune cells. J Mol Biol.

[B67] Ichikawa JK, Norris A, Bandera MG, Geiss GK, van't Wout AB, Bumgarner R, Lory S (2000). Interaction of Pseudomonas aeruginosa with epithelial cells: Identification of differentially regulated genes by expression microarray analysis of human cDNAs. Proc Natl Acad Sci USA.

[B68] Kel AE, Gossling E, Reuter I, Cheremushkin E, Kel-Margoulis OV, Wingender E (2003). MATCH: A tool for searching transcription factor binding sites in DNA sequences. Nucleic Acids Res.

[B69] Davuluri RV, Grosse I, Zhang MQ (2001). Computational identification of promoters and first exons in the human genome. Nat Genet.

[B70] Matys V, Fricke E, Geffers R, Gößling E, Haubrock M, Hehl R, Hornischer K, Karas D, Kel AE, Kel-Margoulis OV, Kloos DU, Land S, Lewicki-Potapov B, Michael H, Münch R, Reuter I, Rotert S, Saxel H, Scheer M, Thiele S, Wingender E (2003). TRANSFAC^® ^: transcriptional regulation, from patterns to profiles. Nucleic Acids Res.

