# Supplementary material for: Construction of predictive promoter models on the example of antibacterial response of human epithelial cells
Source: Theor Biol Med Model. 2005 Jan 12;2:2. doi: 10.1186/1742-4682-2-2 (PMC546226; doi:10.1186/1742-4682-2-2)
Supplement: Additional File 1 — The whole list of genes found with the promoter model when applying it to the collection of 13000 human 5'-upstream sequences. This list is not cleaned from hypothetical genes. [file 1742-4682-2-2-S1.doc]

Potential target genes (whole list)

1. MGC3200 hypothetical protein MGC3200, LocusLink=84265
2. FLJ20277 hypothetical protein FLJ20277, LocusLink=55624
3. POU2F1 POU domain, class 2, transcription factor 1, LocusLink=5451
4. EFNA3 ephrin-A3, LocusLink=1944
5. OAZ3 ornithine decarboxylase antizyme 3, LocusLink=51686
6. FLJ20139 hypothetical protein FLJ20139, LocusLink=54833
7. ARHC ras homolog gene family, member C, LocusLink=389
8. CRABP2 cellular retinoic acid-binding protein 2, LocusLink=1382
9. SIAT6 sialyltransferase 6 (N-acetyllacosaminide alpha 2,3-sialyltransferase)
10. FLJ13181 hypothetical protein FLJ13181, LocusLink=80263
11. TNFRSF14 tumor necrosis factor receptor superfamily, member 14 (herpesvirus entry mediator);
12. NUF2R hypothetical protein NUF2R, LocusLink=83540
13. PTAFR platelet-activating factor receptor, LocusLink=5724
14. LOC57147 hypothetical protein LOC57147, LocusLink=57147
15. GFI1 growth factor independent 1, LocusLink=2672
16. FLJ11220 hypothetical protein FLJ11220, LocusLink=54665
17. KIAA0673 KIAA0673 protein, LocusLink=23128
18. FLJ23323 hypothetical protein FLJ23323, LocusLink=79707
19. CEZANNE zinc finger protein Cezanne, LocusLink=56957
20. KIAA0736 KIAA0736 gene product, LocusLink=9900
21. KIAA0761 Mid-1-related chloride channel 1, LocusLink=23155
22. DD96 epithelial protein up-regulated in carcinoma, membrane associated protein 17
23. DDOST dolichyl-diphosphooligosaccharide-protein glycosyltransferase, LocusLink=1
24. FLJ10349 hypothetical protein FLJ10349, LocusLink=54707
25. FLJ12455 hypothetical protein FLJ12455, LocusLink=63906
26. DKFZP564D0478 hypothetical protein DKFZp564D0478, LocusLink=84065
27. NCF2 neutrophil cytosolic factor 2 (65kD, chronic granulomatous disease, autosomal 2)
28. AP4B1 adaptor-related protein complex 4, beta 1 subunit, LocusLink=10717
29. ASML3B acid sphingomyelinase-like phosphodiesterase, LocusLink=27293
30. CLASPIN homolog of Xenopus Claspin, LocusLink=63967
31. FLJ20435 hypothetical protein FLJ20435, LocusLink=54933
32. ID3 inhibitor of DNA binding 3, dominant negative helix-loop-helix protein
33. LGR6 leucine-rich repeat-containing G protein-coupled receptor 6, LocusLink=59352
34. IL12RB2 interleukin 12 receptor, beta 2, LocusLink=3595TTTAA
35. ZNF265 zinc finger protein 265, LocusLink=9406
36. FCGR3B Fc fragment of IgG, low affinity IIIb, receptor for (CD16), LocusLink=2215
37. HOOK1 hook1 protein, LocusLink=51361
38. FLJ11269 hypothetical protein FLJ11269, LocusLink=55791
39. DKFZp547A023 hypothetical protein DKFZp547A023, LocusLink=55917
40. CNR2 cannabinoid receptor 2 (macrophage), LocusLink=1269
41. CX46.6 connexin46.6, LocusLink=57165
42. FLJ12875 hypothetical protein FLJ12875, LocusLink=79594
43. KIAA1639 KIAA1639 protein, LocusLink=57729
44. FLJ14351 hypothetical protein FLJ14351, LocusLink=79787
45. SLC16A4 solute carrier family 16 (monocarboxylic acid transporters), member 4
46. AMPD2 adenosine monophosphate deaminase 2 (isoform L), LocusLink=271
47. MTHFR 5,10-methylenetetrahydrofolate reductase (NADPH), LocusLink=4524
48. RAP1GA1 RAP1, GTPase activating protein 1, LocusLink=5909
49. PLA2G5 phospholipase A2, group V, LocusLink=5322
50. EIF3S2 eukaryotic translation initiation factor 3, subunit 2 (beta, 36kD)
51. CDA cytidine deaminase, LocusLink=978
52. SSA2 Sjogren syndrome antigen A2 (60kD, ribonucleoprotein autoantigen SS-A/Ro)
53. HPIP hematopoietic PBX-interacting protein, LocusLink=57326
54. MGC10820 hypothetical protein MGC10820, LocusLink=84734
55. RP4-622L5 hypothetical protein RP4-622L5, LocusLink=56063
56. LIECG3 P3ECSL, LocusLink=64129
57. KIAA1353 KIAA1353 protein, LocusLink=57548
58. LAPTM5 Lysosomal-associated multispanning membrane protein-5, LocusLink=7805
59. MCP membrane cofactor protein (CD46, trophoblast-lymphocyte cross-reactive antigen)
60. NPPB natriuretic peptide precursor B, LocusLink=4879
61. LOC84648 epidermal differentiation complex protein like protein, LocusLink=84648
62. KCNK2 potassium channel, subfamily K, member 2 (TREK-1), LocusLink=3776
63. LOC57823 19A24 protein, LocusLink=57823
64. INPP5B inositol polyphosphate-5-phosphatase, 75kD, LocusLink=3633
65. SYCP1 synaptonemal complex protein 1, LocusLink=6847
66. MGC955 hypothetical protein MGC955, LocusLink=79078
67. GROS1 growth suppressor 1, LocusLink=64175
68. GSTM5 glutathione S-transferase M5, LocusLink=2949
69. FLJ22353 hypothetical protein FLJ22353, LocusLink=79639
70. DDR2 discoidin domain receptor family, member 2, LocusLink=4921
71. KIAA0468 KIAA0468 gene product, LocusLink=9672
72. FLJ11280 hypothetical protein FLJ11280, LocusLink=55793
73. COP9 COP9 homolog;, LocusLink=10920
74. LOC51315 hypothetical protein;, LocusLink=51315
75. ATP6B1 ATPase, H+ transporting, lysosomal (vacuolar proton pump), beta polypeptide, 56/58kD
76. BOLL bol (Drosophila boule homolog)-like;, LocusLink=66037
77. HSA242910 N-Acetylglucosamine kinase;, LocusLink=55577
78. MAL mal, T-cell differentiation protein;, LocusLink=4118
79. ADCY3 adenylate cyclase 3;, LocusLink=109
80. CML1 kidney- and liver-specific gene;, LocusLink=9027
81. PAX8 paired box gene 8;, LocusLink=7849
82. GP3ST glycoprotein beta-Gal 3'-sulfotransferase;, LocusLink=64090
83. FLJ14126 hypothetical protein FLJ14126;, LocusLink=79907
84. TGFA transforming growth factor, alpha;, LocusLink=7039
85. CIR CBF1 interacting corepressor;, LocusLink=9541
86. ACVR1 activin A receptor, type I;, LocusLink=90
87. CRYPTIC cryptic gene;, LocusLink=55997
88. PAP pancreatitis-associated protein;, LocusLink=5068
89. PAN2 PAN2 protein;, LocusLink=57665
90. REPRIMO candidate mediator of the p53-dependent G2 arrest;, LocusLink=56475
91. SCYA20 small inducible cytokine subfamily A (Cys-Cys), member 20;, LocusLink=63
92. ZNF142 zinc finger protein 142 (clone pHZ-49);, LocusLink=7701
93. VIL1 villin 1;, LocusLink=7429
94. MGC11104 hypothetical protein MGC11104;, LocusLink=84269
95. IL1HY1 interleukin 1, delta;, LocusLink=26525
96. CASP8 caspase 8, apoptosis-related cysteine protease;, LocusLink=841
97. FLJ14708 hypothetical protein FLJ14708;, LocusLink=84913
98. PRO2900 hypothetical protein PRO2900;, LocusLink=55498
99. FLJ10035 hypothetical protein FLJ10035;, LocusLink=55054
100. MPV17 MpV17 transgene, murine homolog, glomerulosclerosis;, LocusLink=4358
101. MPHOSPH10 M-phase phosphoprotein 10 (U3 small nucleolar ribonucleoprotein);, LocusLink
102. RNF30 ring finger protein 30;, LocusLink=57159
103. KIAA1266 KIAA1266 protein;, LocusLink=57504
104. PIG3 quinone oxidoreductase homolog;, LocusLink=9540
105. MYT1L myelin transcription factor 1-like;, LocusLink=23040
106. VIT1 vitiligo-associated protein VIT-1;, LocusLink=55519
107. CAPG capping protein (actin filament), gelsolin-like;, LocusLink=822
108. MGC12936 hypothetical protein MGC12936;, LocusLink=84276
109. IL12A interleukin 12A (natural killer cell stimulatory factor 1, cytotoxic lymphocyte maturati
110. DKFZP434F091 DKFZP434F091 protein;, LocusLink=25917
111. DKFZP564O123 DKFZP564O123 protein;, LocusLink=25978
112. LOC51095 CGI-47 protein;, LocusLink=51095
113. MRPS25 mitochondrial ribosomal protein S25;, LocusLink=64432
114. FLJ23188 hypothetical protein FLJ23188;, LocusLink=79681
115. FLJ14454 hypothetical protein FLJ14454;, LocusLink=84873
116. GBE1 glucan (1,4-alpha-), branching enzyme 1 (glycogen branching enzyme, Andersen disease, gly
117. MGC10796 hypothetical protein MGC10796;, LocusLink=79413
118. FETUB fetuin B;, LocusLink=26998
119. SRP stresscopin-related peptide;, LocusLink=90226
120. TCTA T-cell leukemia translocation altered gene;, LocusLink=6988
121. JWA vitamin A responsive; cytoskeleton related;, LocusLink=10550
122. FLJ20059 hypothetical protein FLJ20059;, LocusLink=54800
123. ITIH1 inter-alpha (globulin) inhibitor, H1 polypeptide;, LocusLink=3697
124. ATP1B3 ATPase, Na+/K+ transporting, beta 3 polypeptide;, LocusLink=483
125. STAG1 stromal antigen 1;, LocusLink=10274
126. LZTFL1 leucine zipper transcription factor-like 1;, LocusLink=54585
127. RPL24 ribosomal protein L24;, LocusLink=6152
128. FLJ20574 hypothetical protein FLJ20574;, LocusLink=54986
129. WNT5A wingless-type MMTV integration site family, member 5A;, LocusLink=7474
130. MLH1 mutL (E. coli) homolog 1 (colon cancer, nonpolyposis type 2);, LocusLink=4292
131. B3GNT5 UDP-GlcNAc:betaGal beta-1,3-N-acetylglucosaminyltransferase 5;, LocusLink=84002
132. FLJ12729 hypothetical protein FLJ12729;, LocusLink=80012
133. LUZP3 leucine zipper protein 3;, LocusLink=83598
134. FLJ22623 hypothetical protein FLJ22623;, LocusLink=79663
135. FLJ14153 hypothetical protein FLJ14153;, LocusLink=64747
136. KIAA1257 KIAA1257 protein;, LocusLink=57501
137. FLJ20730 hypothetical protein FLJ20730;, LocusLink=55032
138. FLJ20551 hypothetical protein FLJ20551;, LocusLink=54977
139. KIAA0332 KIAA0332 protein;, LocusLink=23350
140. MCCC1 methylcrotonoyl-Coenzyme A carboxylase 1 (alpha);, LocusLink=56922
141. LOC51161 g20 protein;, LocusLink=51161
142. MGC2408 hypothetical protein MGC2408;, LocusLink=84291
143. NFKB1 nuclear factor of kappa light polypeptide gene enhancer in B-cells 1 (p105);
144. KIAA1808 KIAA1808 protein;, LocusLink=84448
145. IRF2 interferon regulatory factor 2;, LocusLink=3660
146. ARHH ras homolog gene family, member H;, LocusLink=399
147. KIAA1239 KIAA1239 protein;, LocusLink=57495
148. KSP37 Ksp37 protein;, LocusLink=83888
149. MAP2K1IP1 mitogen-activated protein kinase kinase 1 interacting protein 1;
150. DDX15 DEAD/H (Asp-Glu-Ala-Asp/His) box polypeptide 15;, LocusLink=1665
151. GRO1 GRO1 oncogene (melanoma growth stimulating activity, alpha);, LocusLink=2919;
152. FLJ11331 hypothetical protein FLJ11331;, LocusLink=55345
153. SCYB5 small inducible cytokine subfamily B (Cys-X-Cys), member 5 (epithelial-derived neutrophi
154. UBE2D3 ubiquitin-conjugating enzyme E2D 3 (homologous to yeast UBC4/5);, LocusLink=732
155. GLRA3 glycine receptor, alpha 3;, LocusLink=8001
156. EGF epidermal growth factor (beta-urogastrone);, LocusLink=1950
157. DKFZP434N1235 hypothetical protein DKFZp434N1235;, LocusLink=83447
158. RPL34 ribosomal protein L34 , LocusLink=6164
159. DKFZP434L1717 hypothetical protein DKFZp434L1717;, LocusLink=84076
160. WHSC2 Wolf-Hirschhorn syndrome candidate 2;, LocusLink=7469
161. FLJ23056 hypothetical protein FLJ23056;, LocusLink=79633
162. RAB28 RAB28, member RAS oncogene family;, LocusLink=9364
163. EREG epiregulin;, LocusLink=2069
164. FLJ10846 hypothetical protein FLJ10846;, LocusLink=55751
165. FLJ10858 hypothetical protein FLJ10858;, LocusLink=55247
166. SEC3 Sec3-like;, LocusLink=55763
167. SLC34A2 solute carrier family 34 (sodium phosphate), member 2, LocusLink=1
168. FLJ20273 hypothetical protein;, LocusLink=54502
169. ZNF141 zinc finger protein 141 (clone pHZ-44);, LocusLink=7700
170. NUP54 nucleoporin p54;, LocusLink=53371
171. IL8 interleukin 8;, LocusLink=3576
172. KIAA0171 KIAA0171 gene product;, LocusLink=9685
173. IL12B interleukin 12B (natural killer cell stimulatory factor 2, cytotoxic lymphocyte maturati
174. DCTN4 dynactin 4 (p62);, LocusLink=51164
175. FLJ10141 hypothetical protein FLJ10141;, LocusLink=55078
176. GPRK6 G protein-coupled receptor kinase 6;, LocusLink=2870
177. OSMR oncostatin M receptor;, LocusLink=9180
178. SLC1A3 solute carrier family 1 (glial high affinity glutamate transporter), member 3;
179. FLJ20125 hypothetical protein FLJ20125;, LocusLink=54826
180. KIAA0433 KIAA0433 protein;, LocusLink=23262
181. NAF1 Nef-associated factor 1;, LocusLink=10318
182. FLJ10290 hypothetical protein FLJ10290;, LocusLink=55696
183. KIAA1029 synaptopodin;, LocusLink=11346
184. P4HA2 procollagen-proline, 2-oxoglutarate 4-dioxygenase (proline 4-hydroxylase), alpha polypep
185. KIAA0372 KIAA0372 gene product;, LocusLink=9652
186. IL13 interleukin 13;, LocusLink=3596
187. CSF2 colony stimulating factor 2 (granulocyte-macrophage);, LocusLink=1437
188. NOLA2 nucleolar protein family A, member 2 (H/ACA small nucleolar RNPs);, LocusLink=55
189. ODZ2 odd Oz/ten-m homolog 2 (Drosophila, mouse);, LocusLink=57451
190. MRPS36 mitochondrial ribosomal protein S36;, LocusLink=92259
191. PCDHGA11 protocadherin gamma subfamily A, 11;, LocusLink=56105
192. TAF2F TATA box binding protein (TBP)-associated factor, RNA polymerase II, F, 55kD;
193. PRIM2A primase, polypeptide 2A (58kD);, LocusLink=5558
194. H2BFA H2B histone family, member A;, LocusLink=8339
195. BCKDHB branched chain keto acid dehydrogenase E1, beta polypeptide (maple syrup urine disease)
196. NDR serine threonine protein kinase;, LocusLink=11329
197. KIAA1599 KIAA1599 protein;, LocusLink=57699
198. FLJ20337 hypothetical protein FLJ20337;, LocusLink=55633
199. KIAA0082 KIAA0082 protein;, LocusLink=23070
200. RCL putative c-Myc-responsive;, LocusLink=10591
201. NOTCH4 Notch (Drosophila) homolog 4;, LocusLink=4855
202. LOC54516 similar to prokaryotic-type class I peptide chain release factors;, LocusLink
203. PTD013 PTD013 protein;, LocusLink=51389
204. SPR1 SPR1 protein;, LocusLink=29112
205. TCF21 transcription factor 21;, LocusLink=6943
206. G6E putative Ly-6 superfamily member;, LocusLink=79136
207. EDN1 endothelin 1;, LocusLink=1906
208. MICB MHC class I polypeptide-related sequence B;, LocusLink=4277
209. BAT8 HLA-B associated transcript 8;, LocusLink=10919
210. BAT4 HLA-B associated transcript 4;, LocusLink=7918
211. HTR1B 5-hydroxytryptamine (serotonin) receptor 1B;, LocusLink=3351
212. BF B-factor, properdin;, LocusLink=629
213. SERPINB1 serine (or cysteine) proteinase inhibitor, clade B (ovalbumin), member 1;
214. DKFZP586D2223 DKFZP586D2223 protein;, LocusLink=25862
215. LOC51323 hypothetical protein;, LocusLink=51323
216. FLJ13693 hypothetical protein FLJ13693;, LocusLink=79865
217. ABT1 TATA-binding protein-binding protein;, LocusLink=29777
218. DKFZP564B116 DKFZP564B116 protein;, LocusLink=25998
219. CLIC5 chloride intracellular channel 5;, LocusLink=53405
220. FLJ23407 hypothetical protein FLJ23407;, LocusLink=79764
221. REV3L REV3 (yeast homolog)-like, catalytic subunit of DNA polymerase zeta;
222. MGC3077 hypothetical protein MGC3077;, LocusLink=79017
223. STK31 serine/threonine kinase 31;, LocusLink=56164
224. NOH61 putative nucleolar RNA helicase;, LocusLink=54606
225. CUTL1 cut (Drosophila)-like 1 (CCAAT displacement protein);, LocusLink=1523
226. CALN1 calneuron 1;, LocusLink=83698
227. TFPI2 tissue factor pathway inhibitor 2;, LocusLink=7980
228. TAC1 tachykinin, precursor 1 (substance K, substance P, neurokinin 1, neurokinin 2, neuromedin
229. LOC51024 CGI-135 protein;, LocusLink=51024
230. HIC I-mfa domain-containing protein;, LocusLink=29969
231. LKR/SDH lysine-ketoglutarate reductase /saccharopine dehydrogenase;, LocusLink=10157;
232. FLJ10900 hypothetical protein FLJ10900;, LocusLink=55253
233. FLJ13195 hypothetical protein FLJ13195 similar to stromal antigen 3;, LocusLink=64940;
234. PAX4 paired box gene 4;, LocusLink=5078
235. ZNF212 zinc finger protein 212;, LocusLink=7988
236. DFNA5 deafness, autosomal dominant 5;, LocusLink=1687
237. AKR1D1 aldo-keto reductase family 1, member D1 (delta 4-3-ketosteroid-5-beta-reductase); chrom
238. HSPC216 hypothetical protein;, LocusLink=51530
239. NDUFB2 NADH dehydrogenase (ubiquinone) 1 beta subcomplex, 2 (8kD, AGGG);
240. NPD007 NPD007 protein;, LocusLink=57414
241. EN2 engrailed homolog 2;, LocusLink=2020
242. HTR5A 5-hydroxytryptamine (serotonin) receptor 5A;, LocusLink=3361
243. FLJ10477 hypothetical protein FLJ10477;, LocusLink=55145
244. KIAA0196 KIAA0196 gene product;, LocusLink=9897
245. TSLRP testis specific leucine rich repeat protein;, LocusLink=23639
246. WISP1 WNT1 inducible signaling pathway protein 1;, LocusLink=8840
247. FLJ22252 hypothetical protein FLJ22252 similar to SRY-box containing gene 17;
248. FOG2 Friend of GATA2;, LocusLink=23414
249. RPS20 ribosomal protein S20;, LocusLink=6224
250. RAD21 RAD21 (S. pombe) homolog;, LocusLink=5885
251. D8S2298E reproduction 8;, LocusLink=7993
252. HT002 HT002 protein; hypertension-related calcium-regulated gene;, LocusLink=28991
253. PNOC prepronociceptin;, LocusLink=5368
254. DLGAP2 discs, large (Drosophila) homolog-associated protein 2;, LocusLink=9228
255. DEFB2 defensin, beta 2;, LocusLink=1673
256. INSL4 insulin-like 4 (placenta);, LocusLink=3641
257. MGC10999 hypothetical protein MGC10999;, LocusLink=84267
258. FLJ10110 hypothetical protein FLJ10110;, LocusLink=55071
259. RGS3 regulator of G-protein signalling 3;, LocusLink=5998
260. FLJ13163 hypothetical protein FLJ13163;, LocusLink=64768
261. ROR2 receptor tyrosine kinase-like orphan receptor 2;, LocusLink=4920
262. MTAP methylthioadenosine phosphorylase;, LocusLink=4507
263. UNC13 UNC13 (C. elegans)-like;, LocusLink=10497
264. FLJ10262 hypothetical protein FLJ10262;, LocusLink=55958
265. FLJ10867 hypothetical protein FLJ10867;, LocusLink=55755
266. C9orf9 chromosome 9 open reading frame 9;, LocusLink=11092
267. DNAI1 dynein, axonemal, intermediate polypeptide, 1;, LocusLink=27019
268. C9orf5 chromosome 9 open reading frame 5;, LocusLink=23731
269. BRD3 bromodomain-containing 3;, LocusLink=8019
270. CD72 CD72 antigen;, LocusLink=971
271. TRAF1 TNF receptor-associated factor 1;, LocusLink=7185
272. KIAA0375 KIAA0375 gene product;, LocusLink=9853
273. FPGS folylpolyglutamate synthase;, LocusLink=2356
274. RXRA retinoid X receptor, alpha;, LocusLink=6256
275. HSPC043 HSPC043 protein;, LocusLink=58493
276. ZID zinc finger protein with interaction domain;, LocusLink=10773
277. ENG endoglin (Osler-Rendu-Weber syndrome 1);, LocusLink=2022
278. FLJ11726 hypothetical protein FLJ11726;, LocusLink=80036
279. KIAA0354 KIAA0354 gene product;, LocusLink=9925
280. MGC14439 hypothetical protein MGC14439, LocusLink=84991
281. RENT2 regulator of nonsense transcripts 2, LocusLink=26019
282. NOLC1 nucleolar and coiled-body phosphprotein 1, LocusLink=9221
283. CSPG6 chondroitin sulfate proteoglycan 6 (bamacan), LocusLink=9126
284. FER1L3 fer-1 (C.elegans)-like 3 (myoferlin), LocusLink=26509
285. FLJ14547 hypothetical protein FLJ14547, LocusLink=84890
286. KIAA1274 KIAA protein (similar to mouse paladin), LocusLink=27143
287. TIM23 translocase of inner mitochondrial membrane 23 (yeast) homolog, LocusLink=1043
288. FLJ10889 hypothetical protein FLJ10889, LocusLink=55760
289. PLA2G13 group XIII secreted phospholipase A2, LocusLink=84647
290. BUB3 BUB3 (budding uninhibited by benzimidazoles 3, yeast) homolog, LocusLink=918
291. PYCS pyrroline-5-carboxylate synthetase (glutamate gamma-semialdehyde synthetase)
292. CYP26A1 cytochrome P450, subfamily XXVIA, polypeptide 1, LocusLink=1592
293. NDUFB8 NADH dehydrogenase (ubiquinone) 1 beta subcomplex, 8 (19kD, ASHI)
294. TNFRSF6 tumor necrosis factor receptor superfamily, member 6, LocusLink=355
295. DEPP decidual protein induced by progesterone, LocusLink=11067
296. SEMA4G sema domain, immunoglobulin domain (Ig), transmembrane domain (TM) and short cytoplasmi
297. CPN1 carboxypeptidase N, polypeptide 1, 50kD, LocusLink=1369
298. BMI1 murine leukemia viral (bmi-1) oncogene homolog, LocusLink=648
299. FLJ20154 hypothetical protein FLJ20154, LocusLink=54838
300. PARG poly (ADP-ribose) glycohydrolase, LocusLink=8505
301. NUMA1 nuclear mitotic apparatus protein 1, LocusLink=4926
302. CHORDC1 cysteine and histidine-rich domain (CHORD)-containing, zinc-binding protein 1
303. FLI1 Friend leukemia virus integration 1, LocusLink=2313
304. MSP mosaic serine protease, LocusLink=84000
305. HSPC138 hypothetical protein, LocusLink=51501
306. EEF1G eukaryotic translation elongation factor 1 gamma, LocusLink=1937
307. TMPRSS5 transmembrane protease, serine 5 (spinesin), LocusLink=80975
308. EHF ets homologous factor, LocusLink=26298
309. MGC10966 hypothetical protein MGC10966, LocusLink=83706
310. FLJ21845 hypothetical protein FLJ21845, LocusLink=79796
311. CLN2 ceroid-lipofuscinosis, neuronal 2, late infantile (Jansky-Bielschowsky disease)
312. SLC22A1L solute carrier family 22 (organic cation transporter), member 1-like
313. NDUFC2 NADH dehydrogenase (ubiquinone) 1, subcomplex unknown, 2 (14.5kD, B14.5b)
314. TRIM5 tripartite motif-containing 5, LocusLink=85363
315. KCNK7 potassium channel, subfamily K, member 7, LocusLink=10089
316. FEN1 flap structure-specific endonuclease 1, LocusLink=2237
317. VPS11 vacuolar protein sorting 11 (yeast homolog), LocusLink=55823
318. KIAA0056 KIAA0056 protein, LocusLink=23310
319. DLG2 discs, large (Drosophila) homolog 2 (chapsyn-110) (NOTE: redefinition of symbol)
320. APOC3 apolipoprotein C-III, LocusLink=345
321. FLJ14427 hypothetical protein FLJ14427, LocusLink=84867
322. MGC2574 hypothetical protein MGC2574, LocusLink=79080
323. APOA4 apolipoprotein A-IV, LocusLink=337
324. CASP4 caspase 4, apoptosis-related cysteine protease, LocusLink=837
325. SDHD succinate dehydrogenase complex, subunit D, integral membrane protein
326. MAP3K11 mitogen-activated protein kinase kinase kinase 11, LocusLink=4296
327. KIAA0088 KIAA0088 protein, LocusLink=23193
328. PME-1 protein phosphatase methylesterase-1, LocusLink=51400
329. KIAA1534 KIAA1534 protein, LocusLink=57656
330. HSMDPKIN myotonic dystrophy protein kinase like protein, LocusLink=55561
331. ASCL2 achaete-scute complex (Drosophila) homolog-like 2, LocusLink=430
332. COX8 cytochrome c oxidase subunit VIII, LocusLink=1351
333. UPK2 uroplakin 2, LocusLink=7379
334. TIMM8B translocase of inner mitochondrial membrane 8 (yeast) homolog B
335. BLR1 Burkitt lymphoma receptor 1, GTP-binding protein, LocusLink=643
336. SLC4A8 solute carrier family 4, sodium bicarbonate cotransporter, member 8
337. HOXC5 homeo box C5, LocusLink=3222
338. KCNA5 potassium voltage-gated channel, shaker-related subfamily, member 5
339. KCNA1 potassium voltage-gated channel, shaker-related subfamily, member 1 (episodic ataxia wit
340. FLJ12448 hypothetical protein FLJ12448, LocusLink=64897
341. DKFZP434D0127 hypothetical protein DKFZp434D0127, LocusLink=84101
342. GIT2 G protein-coupled receptor kinase-interactor 2, LocusLink=9815
343. IRAK-M interleukin-1 receptor-associated kinase M, LocusLink=11213
344. LOC51729 Npw38-binding protein NpwBP, LocusLink=51729
345. FLJ12750 hypothetical protein FLJ12750, LocusLink=79720
346. C2F putative protein, LocusLink=10436
347. KIAA0537 KIAA0537 gene product, LocusLink=9891
348. ADMR adrenomedullin receptor, LocusLink=11318
349. AAAS achalasia, adrenocortical insufficiency, alacrimia (Allgrove, triple-A)
350. PTPRR protein tyrosine phosphatase, receptor type, R, LocusLink=5801
351. OAS1 2',5'-oligoadenylate synthetase 1 (40-46 kD), LocusLink=4938
352. LOC51290 CDA14, LocusLink=51290
353. PPFIBP1 PTPRF interacting protein, binding protein 1 (liprin beta 1), LocusLink=8496
354. IL23A interleukin 23, alpha subunit p19, LocusLink=51561
355. CRBPIII putative cellular retinol-binding protein CRBP III, LocusLink=83758
356. RFX4 regulatory factor X, 4 (influences HLA class II expression), LocusLink=5992
357. NCOR2 nuclear receptor co-repressor 2, LocusLink=9612
358. LUM lumican, LocusLink=4060
359. P11 protease, serine, 22, LocusLink=8909
360. UBE2N ubiquitin-conjugating enzyme E2N (homologous to yeast UBC13), LocusLink=7334;
361. AQP2 aquaporin 2 (collecting duct), LocusLink=359
362. FLJ13491 hypothetical protein FLJ13491, LocusLink=79676
363. RAB5B RAB5B, member RAS oncogene family, LocusLink=5869
364. PP1057 hypothetical protein PP1057, LocusLink=83441
365. TARBP2 TAR (HIV) RNA-binding protein 2, LocusLink=6895
366. MLL2 myeloid/lymphoid or mixed-lineage leukemia 2, LocusLink=8085
367. GALNT4 UDP-N-acetyl-alpha-D-galactosamine:polypeptide N-acetylgalactosaminyltransferase 4 (Gal
368. P85SPR PAK-interacting exchange factor beta, Locuslink=8874
369. KIAA1165 hypothetical protein KIAA1165, LocusLink=54602
370. HSP105B heat shock 105kD. LocusLink=10808
371. MAB21L1 mab-21 (c.elegans)-like 1, LocusLink=4081
372. RNF17 ring finger protein 17, LocusLink=56163
373. KIAA1822 KIAA1822 protein, LocusLink=84439
374. FLJ22558 hypothetical protein FLJ22558, LocusLink=64758
375. FLJ20644 hypothetical protein FLJ20644, LocusLink=55012
376. KCNK10 potassium channel, subfamily K, member 10 (TREK-2), LocusLink=54207
377. KIAA0391 KIAA0391 gene product, LocusLink=9692
378. FOXG1B forkhead box G1B, LocusLink=2290
379. ADPRTL2 ADP-ribosyltransferase (NAD+; poly(ADP-ribose) polymerase)-like 2
380. TLH29 TLH29 protein precursor, LocusLink=83982
381. PLPL myelin proteolipid protein-like protein, LocusLink=56936
382. CEBPE CCAAT/enhancer binding protein (C/EBP), epsilon, LocusLink=1053
383. LOC85439 stonin 2, LocusLink=85439
384. MGC12435 hypothetical protein MGC12435, LocusLink=83544
385. FLJ20371 hypothetical protein FLJ20371, LocusLink=55640
386. CIDEB cell death-inducing DFFA-like effector b, LocusLink=27141
387. KIAA0317 KIAA0317 gene product, LocusLink=9870
388. BATF basic leucine zipper transcription factor, ATF-like, LocusLink=10538
389. GTF2A1 general transcription factor IIA, 1 (37kD and 19kD subunits), LocusLink=2957;
390. EIF5 eukaryotic translation initiation factor 5, LocusLink=1983
391. PNN pinin, desmosome associated protein, LocusLink=5411
392. TINF2 TERF1 (TRF1)-interacting nuclear factor 2, LocusLink=26277
393. FLJ10111 hypothetical protein FLJ10111, LocusLink=55072
394. D15S226E Prader-Willi/Angelman syndrome-5, LocusLink=8123
395. LOC51049 clone 1900 unknown protein, LocusLink=51049
396. FLJ23168 hypothetical protein FLJ23168, LocusLink=80125
397. ISLR immunoglobulin superfamily containing leucine-rich repeat, LocusLink=367
398. CYP19 cytochrome P450, subfamily XIX (aromatization of androgens), LocusLink=1588
399. IGF1R insulin-like growth factor 1 receptor, LocusLink=3480
400. ITGA11 integrin, alpha 11, LocusLink=22801
401. HCN4 hyperpolarization activated cyclic nucleotide-gated potassium channel 4
402. MDS009 x 009 protein, LocusLink=56986
403. GTF2A2 general transcription factor IIA, 2 (12kD subunit), LocusLink=2958
404. MGC14386 similar to cyclin-E binding protein 1 (H. sapiens), LocusLink=91433
405. KNSL5 kinesin-like 5 (mitotic kinesin-like protein 1), LocusLink=9493
406. FLJ12973 hypothetical protein FLJ12973, LocusLink=79968DKFZp434J1815 hypothetical protein DKFZp434J1815, LocusLink=84204
407. FLJ20040 hypothetical protein, LocusLink=54442
408. CRYM crystallin, mu, LocusLink=1428
409. PARD6A par-6 (partitioning defective 6, C.elegans) homolog alpha
410. ORC6L origin recognition complex, subunit 6 (yeast homolog)-like, LocusLink=23594; 2
411. KIAA1784 KIAA1784 protein, LocusLink=84464
412. SULT1A1 sulfotransferase family, cytosolic, 1A, phenol-preferring, member 1
413. KIAA0326 KIAA0326 protein, LocusLink=23361
414. KIAA1348 KIAA1348 protein, LocusLink=57546
415. FLJ13111 hypothetical protein FLJ13111, LocusLink=80152
416. RCD-8 autoantigen, LocusLink=23644
417. GNAO1 guanine nucleotide binding protein (G protein), alpha activating activity polypeptide O;
418. PLK polo (Drosophia)-like kinase, LocusLink=5347
419. HSPC171 HSPC171 protein, LocusLink=29100
420. PRO0461 PRO0461 protein, LocusLink=28993
421. KIAA0399 KIAA0399 protein, LocusLink=23140
422. GK001 GK001 protein, LocusLink=57003
423. MGC2963 hypothetical protein MGC2963, LocusLink=83460
424. TIP-1 Tax interaction protein 1, LocusLink=30851
425. SOX20 SRY (sex determining region Y)-box 20, LocusLink=6665
426. NAGLU N-acetylglucosaminidase, alpha- (Sanfilippo disease IIIB), LocusLink=46
427. HT008 uncharacterized hypothalamus protein HT008, LocusLink=55852
428. SLC4A1 solute carrier family 4, anion exchanger, member 1 (erythrocyte membrane protein band 3
429. FLJ14069 hypothetical protein FLJ14069, LocusLink=80091
430. RDGBB retinal degeneration B beta, LocusLink=26207
431. IFI35 interferon-induced protein 35, LocusLink=3430
432. AD023 AD023 protein, LocusLink=57409
433. USP22 ubiquitin specific protease 22, LocusLink=23326
434. LAK-4P expressed in activated T/LAK lymphocytes, LocusLink=11322
435. UBB ubiquitin B, LocusLink=7314
436. ARHGDIA Rho GDP dissociation inhibitor (GDI) alpha, LocusLink=396
437. VTS58635 ras-like protein VTS58635, LocusLink=91608
438. HSY11339 GalNAc alpha-2, 6-sialyltransferase I, long form, LocusLink=55808
439. FLJ22341 hypothetical protein FLJ22341, LocusLink=79651
440. MPP3 membrane protein, palmitoylated 3 (MAGUK p55 subfamily member 3)
441. HCNGP transcriptional regulator protein, LocusLink=29115
442. ZNF179 zinc finger protein 179, LocusLink=7732
443. FLJ20721 hypothetical protein FLJ20721, LocusLink=55028
444. HOXB4 homeo box B4, LocusLink=3214
445. HOXB2 homeo box B2, LocusLink=3212
446. SCYA23 small inducible cytokine subfamily A (Cys-Cys), member 23, LocusLink=636
447. NUP88 nucleoporin 88kD, LocusLink=4927
448. RECQL5 RecQ protein-like 5, LocusLink=9400
449. GALR2 galanin receptor 2, LocusLink=8811
450. KSR kinase suppressor of ras, LocusLink=8844
451. LOC56270 hypothetical protein 628, LocusLink=56270
452. EPX eosinophil peroxidase, LocusLink=8288
453. SCYA11 small inducible cytokine subfamily A (Cys-Cys), member 11 (eotaxin)
454. AOC3 amine oxidase, copper containing 3 (vascular adhesion protein 1), LocusLink=
455. VATI membrane protein of cholinergic synaptic vesicles, LocusLink=10493
456. SOST sclerostin, LocusLink=50964
457. WNT3 wingless-type MMTV integration site family, member 3, LocusLink=7473
458. MGC3123 hypothetical protein MGC3123, LocusLink=79089
459. HML2 macrophage lectin 2 (calcium dependent), LocusLink=10462
460. GALK1 galactokinase 1, LocusLink=2584
461. SFRS2 splicing factor, arginine/serine-rich 2, LocusLink=6427
462. VTN vitronectin (serum spreading factor, somatomedin B, complement S-protein)
463. ZNF24 zink finger protein 24 (KOX17), LocusLink=84307
464. RNMT RNA (guanine –7-)methyltransferase, LocusLink=8731
465. MC2R melanocortin 2 receptor, LocusLink=4158
466. B29 B29protein, LocusLink=8876
467. BRUNOL4 Bruno (Drosophila)-like 4, RNA binding protein, LocusLink=56853
468. SNRPD1 smallnuclear ribonucleoprotein D1 polypeptide (16kD), LocusLink=6632
469. ELAC1 elaC (E.coli) homolog 1, LocusLink=55520
470. EDG5 endothelial differentiation, sphingolipid G-protein-coupled receptor, 5
471. GIOT-2 GIOT-2 for gonadotropin inducible transcription repressor-2, LocusLink=51710;
472. CD209L CD209 antigen-like, LocusLink=10332
473. KIAA1776 fibrillin3, LocusLink=84467
474. SIRT6 sirtuin (silent mating type information regulation 2, S. cerevisiae, homolog) 6
475. SIRT2 sirtuin (silent mating type information regulation 2, S.cerevisiae, homolog) 2
476. CD37 CD37 antigen, LocusLink=951
477. AKT2 v-akt murine thymoma viral oncogene homolog 2, LocusLink=208
478. MCC2 AIE-75 binding protein protein, LocusLink=83878
479. MGC2747 hypothetical protein MGC2747, LocusLink=79086
480. FLJ21742 hypothetical protein FLJ21742, LocusLink=84167
481. CEACAM4 carcinoembryonic antigen-related cell adhesion molecule 4, LocusLink=10
482. CEACAM3 carcinoembryonic antigen-related cell adhesion molecule 3, LocusLink=10
483. CDC37 CDC37 (cell division cycle 37, S. cerevisiae, homolog), LocusLink=11140
484. FLJ22187 hypothetical protein FLJ22187, LocusLink=80207
485. COL5A3 collagen, type V, alpha 3, LocusLink=50509
486. CYP4F11 cytochrome P450, subfamily IVF, polypeptide 11, LocusLink=57834
487. IRF3 interferon regulatory factor 3, LocusLink=3661
488. BC-2 putative breast adenocarcinoma marker (32kD), LocusLink=27243
489. TNFSF7 tumor necrosis factor (ligand) superfamily, member 7, LocusLink=970
490. NSP1 novel SH2-containing protein 1, LocusLink=10045
491. MGC10974 hypothetical protein MGC10974, LocusLink=84266
492. FLJ22757 hypothetical protein FLJ22757, LocusLink=79958
493. CYP4F2 cytochrome P450, subfamily IVF, polypeptide 2, LocusLink=8529
494. EEF2 eukaryotic translation elongation factor 2, LocusLink=1938
495. BBC3 Bcl-2 binding component 3, LocusLink=27113
496. APOC1 apolipoprotein C-I, LocusLink=341
497. LOC51231 VRK3 for vaccinia related kinase 3, LocusLink=51231
498. INSR insulin receptor, LocusLink=3643
499. EZFIT endothelial zinc finger protein induced by tumor necrosis factor alpha
500. KLK4 kallikrein 4 (prostase, enamel matrix, prostate), LocusLink=9622
501. MGC11271 hypothetical protein MGC11271, LocusLink=79173
502. FLJ23447 hypothetical protein FLJ23447, LocusLink=79883
503. RELB v-rel avian reticuloendotheliosis viral oncogene homolog B (nuclear factor of kappa light
504. KLK1 kallikrein 1, renal/pancreas/salivary, LocusLink=3816
505. ACP5 acid phosphatase 5, tartrate resistant, LocusLink=54
506. LOC91120 similar to ZINC FINGER PROTEIN 85 (ZINC FINGER PROTEIN HPF4) (HTF1) (H. sapiens); chr
507. ZNF136 zinc finger protein 136 (clone pHZ-20), LocusLink=7695
508. MGC11314 hypothetical protein MGC11314, LocusLink=84807
509. DKFZP564A1164 hypothetical protein DKFZp564A1164, LocusLink=84063
510. TZFP testis zinc finger protein, LocusLink=27033
511. ICAM3 intercellular adhesion molecule 3, LocusLink=3385
512. ICAM1 intercellular adhesion molecule 1 (CD54), human rhinovirus receptor
513. LILRB5 leukocyte immunoglobulin-like receptor, subfamily B (with TM and ITIM domains), member
514. LILRB4 leukocyte immunoglobulin-like receptor, subfamily B (with TM and ITIM domains), member
515. LILRB3 leukocyte immunoglobulin-like receptor, subfamily B (with TM and ITIM domains), member
516. PNKP polynucleotide kinase 3'-phosphatase, LocusLink=11284
517. FLJ11286 hypothetical protein FLJ11286, LocusLink=55337
518. GP6 glycoprotein VI (platelet), LocusLink=51206
519. ZNF226 zinc finger protein 226, LocusLink=7769
520. ZNF222 zinc finger protein 222, LocusLink=7673
521. MBC3205 hypothetical protein MBC3205, LocusLink=90585
522. PPP1R15A protein phosphatase 1, regulatory (inhibitor) subunit 15A, LocusLink=2
523. NFKBIB nuclear factor of kappa light polypeptide gene enhancer in B-cells inhibitor, beta; chr
524. LENG4 leukocyte receptor cluster (LRC) member 4, LocusLink=79143
525. GPR4 G protein-coupled receptor 4, LocusLink=2828
526. KIAA1769 KIAA1769 protein;, LocusLink=85441
527. PMP24 24 kDa intrinsic membrane protein, LocusLink=11264
528. CHGB chromogranin B (secretogranin 1), LocusLink=1114
529. GMEB2 glucocorticoid modulatory element binding protein 2, LocusLink=26205
530. DPM1 dolichyl-phosphate mannosyltransferase polypeptide 1, catalytic subunit
531. FLJ22376 hypothetical protein FLJ22376; , LocusLink=64773
532. LOC51605 CGI-09 protein; , LocusLink=51605
533. SGK2 serum/glucocorticoid regulated kinase 2; , LocusLink=10110
534. PTPN1 protein tyrosine phosphatase, non-receptor type 1, LocusLink=5770;
535. ADF destrin (actin depolymerizing factor), LocusLink=11034
536. RPC39 polymerase (RNA) III (DNA directed) (39kD), LocusLink=10621
537. ARFRP1 ADP-ribosylation factor related protein 1, LocusLink=10139
538. TUBB1 beta tubulin 1, class VI, LocusLink=81027
539. MGC4294 hypothetical protein MGC4294 , LocusLink=79160
540. DLM1 tumor stroma and activated macrophage protein DLM-1 , LocusLink=81030
541. FLJ22504 hypothetical C2H2 zinc finger protein FLJ22504 , LocusLink=63925
542. CLG01 clg01 protein , LocusLink=27296
543. HSPC207 hypothetical protein , LocusLink=51526
544. LOC55902 acetyl-CoA synthetase; , LocusLink=55902
545. TMPRSS3 transmembrane rpotease, serine 3, LocusLink=64699
546. CRYAA crystallin alpha A, LocusLink=1409
547. COL6A2 collagen, type VI, alpha2, LocusLink=1292
548. CBR1 carbonyl reductase 1, LocusLink=873
549. ATP50 ATP synthase, H^+^ transporting, mitochndrial F1 complex, O subunit
550. GALR3 galanin receptor 3, LocusLink=8484
551. PLA2G6 phospholipase A2, group VI (cytosolic, calcium-independent), LocusLink=8
552. DKFZP761I141 hypothetical protein DKFZp761I141, LocusLink=83746
553. KIAA1655 KIAA1655 protein, LocusLink=85370
554. RFPL2 ret finger protein-like 2, LocusLink=10739
555. RFPL1 ret finger protein-like 1, LocusLink=5988
556. CTRP6 complement-c1q tumor necrosis factor-related protein 6, LocusLink=83847
557. FLJ12242 hypothetical protein FLJ12242, LocusLink=79734
558. TCN2 transcobalamin II; macrocytic anemia, LocusLink=6948
559. C22orf5 chromosome 22 open reading frame 5, LocusLink=25829
560. EIF3S7 eukaryotic translation initiation factor 3, subunit 7 (zeta, 66/67kD)
561. PPARA peroxisome proliferative activated receptor, alpha, LocusLink=5465; 2
562. DNAL4 dynein, axonemal, light polypeptide 4, LocusLink=10126
563. KIAA1364 KIAA1364 protein, LocusLink=57553
564. RBM9 RNA binding motif protein 9, LocusLink=23543
565. VPREB3 pre-B lymphocyte gene 3, LocusLink=29802
566. ARSE arylsulfatase E (chondrodysplasia punctata 1), LocusLink=415
567. SOX3 SRY (sex determining region Y)-box 3, LocusLink=6658
568. RBM10 RNA binding motif protein 10, LocusLink=8241
569. RPS4X ribosomal protein S4, X-linked, LocusLink=6191
570. APR-1 APR-1 protein, LocusLink=28986
571. LOC51634 CGI-79 protein, LocusLink=51634
572. APEXL2 apurinic/apyrimidinic endonuclease(APEX nuclease)-like 2 protein, LocusLink=27
573. RPL44 ribosomal protein L44, LocusLink=6173
574. FLJ20494 similar to mouse neuronal protein 15.6, LocusLink=54539
575. SLC35A2 solute carrier family 35 (UDP-galactose transporter), member 2
576. IKBKG inhibitor of kappa light polypeptide gene enhancer in B-cells, kinase gamma
577. ARAF1 v-raf murine sarcoma 3611 viral oncogene homolog 1, LocusLink=369;
578. DKFZP586N0819 DKFZP586N0819 protein, LocusLink=25899
579. ELK1 ELK1, member of ETS oncogene family, LocusLink=2002
580. FLJ14084 hypothetical protein FLJ14084, LocusLink=59353
581. SLC6A14 solute carrier family 6 (neurotransmitter transporter), member 14
582. RBMY1A1 RNA binding motif protein
