# Supplementary material for: Construction of predictive promoter models on the example of antibacterial response of human epithelial cells
Source: Theor Biol Med Model. 2005 Jan 12;2:2. doi: 10.1186/1742-4682-2-2 (PMC546226; doi:10.1186/1742-4682-2-2)
Supplement: Additional File 2 — The list of genes (found with the promoter model when applying it to the collection of 13000 human 5'-upstream sequences) cleaned from hypothetical genes. [file 1742-4682-2-2-S2.doc]

Potential target genes (cleaned from hypothetical products)

1. POU2F1 POU domain, class 2, transcription factor 1, LocusLink=5451
2. EFNA3 ephrin-A3, LocusLink=1944
3. OAZ3 ornithine decarboxylase antizyme 3, LocusLink=51686
4. ARHC ras homolog gene family, member C, LocusLink=389
5. CRABP2 cellular retinoic acid-binding protein 2, LocusLink=1382
6. SIAT6 sialyltransferase 6 (N-acetyllacosaminide alpha 2,3-sialyltransferase)
7. TNFRSF14 tumor necrosis factor receptor superfamily, member 14 (herpesvirus entry mediator);
8. PTAFR platelet-activating factor receptor, LocusLink=5724
9. GFI1 growth factor independent 1, LocusLink=2672
10. CEZANNE zinc finger protein Cezanne, LocusLink=56957
11. DD96 epithelial protein up-regulated in carcinoma, membrane associated protein 17
12. DDOST dolichyl-diphosphooligosaccharide-protein glycosyltransferase, LocusLink=1
13. NCF2 neutrophil cytosolic factor 2 (65kD, chronic granulomatous disease, autosomal 2)
14. AP4B1 adaptor-related protein complex 4, beta 1 subunit, LocusLink=10717
15. ASML3B acid sphingomyelinase-like phosphodiesterase, LocusLink=27293
16. CLASPIN homolog of Xenopus Claspin, LocusLink=63967
17. ID3 inhibitor of DNA binding 3, dominant negative helix-loop-helix protein
18. LGR6 leucine-rich repeat-containing G protein-coupled receptor 6, LocusLink=59352
19. IL12RB2 interleukin 12 receptor, beta 2, LocusLink=3595TTTAA
20. ZNF265 zinc finger protein 265, LocusLink=9406
21. FCGR3B Fc fragment of IgG, low affinity IIIb, receptor for (CD16), LocusLink=2215
22. HOOK1 hook1 protein, LocusLink=51361
23. CNR2 cannabinoid receptor 2 (macrophage), LocusLink=1269
24. CX46.6 connexin46.6, LocusLink=57165
25. SLC16A4 solute carrier family 16 (monocarboxylic acid transporters), member 4
26. AMPD2 adenosine monophosphate deaminase 2 (isoform L), LocusLink=271
27. MTHFR 5,10-methylenetetrahydrofolate reductase (NADPH), LocusLink=4524
28. RAP1GA1 RAP1, GTPase activating protein 1, LocusLink=5909
29. PLA2G5 phospholipase A2, group V, LocusLink=5322
30. EIF3S2 eukaryotic translation initiation factor 3, subunit 2 (beta, 36kD)
31. CDA cytidine deaminase, LocusLink=978
32. SSA2 Sjogren syndrome antigen A2 (60kD, ribonucleoprotein autoantigen SS-A/Ro)
33. HPIP hematopoietic PBX-interacting protein, LocusLink=57326
34. LIECG3 P3ECSL, LocusLink=64129
35. LAPTM5 Lysosomal-associated multispanning membrane protein-5, LocusLink=7805
36. MCP membrane cofactor protein (CD46, trophoblast-lymphocyte cross-reactive antigen)
37. NPPB natriuretic peptide precursor B, LocusLink=4879
38. LOC84648 epidermal differentiation complex protein like protein, LocusLink=84648
39. KCNK2 potassium channel, subfamily K, member 2 (TREK-1), LocusLink=3776
40. LOC57823 19A24 protein, LocusLink=57823
41. INPP5B inositol polyphosphate-5-phosphatase, 75kD, LocusLink=3633
42. SYCP1 synaptonemal complex protein 1, LocusLink=6847
43. GROS1 growth suppressor 1, LocusLink=64175
44. GSTM5 glutathione S-transferase M5, LocusLink=2949
45. DDR2 discoidin domain receptor family, member 2, LocusLink=4921
46. COP9 COP9 homolog, LocusLink=10920
47. ATP6B1 ATPase, H+ transporting, lysosomal (vacuolar proton pump), beta polypeptide, 56/58kD
48. BOLL bol (Drosophila boule homolog)-like, LocusLink=66037
49. HSA242910 N-Acetylglucosamine kinase, LocusLink=55577
50. MAL mal, T-cell differentiation protein, LocusLink=4118
51. ADCY3 adenylate cyclase 3, LocusLink=109
52. CML1 kidney- and liver-specific gene, LocusLink=9027
53. PAX8 paired box gene 8, LocusLink=7849
54. GP3ST glycoprotein beta-Gal 3'-sulfotransferase, LocusLink=64090
55. TGFA transforming growth factor, alpha, LocusLink=7039
56. CIR CBF1 interacting corepressor, LocusLink=9541
57. ACVR1 activin A receptor, type I, LocusLink=90
58. CRYPTIC cryptic gene, LocusLink=55997
59. PAP pancreatitis-associated protein, LocusLink=5068
60. PAN2 PAN2 protein, LocusLink=57665
61. REPRIMO candidate mediator of the p53-dependent G2 arrest, LocusLink=56475
62. SCYA20 small inducible cytokine subfamily A (Cys-Cys), member 20, LocusLink=63
63. ZNF142 zinc finger protein 142 (clone pHZ-49), LocusLink=7701
64. VIL1 villin 1, LocusLink=7429
65. IL1HY1 interleukin 1, delta, LocusLink=26525
66. CASP8 caspase 8, apoptosis-related cysteine protease, LocusLink=841
67. MPV17 MpV17 transgene, murine homolog, glomerulosclerosis, LocusLink=4358
68. MPHOSPH10 M-phase phosphoprotein 10 (U3 small nucleolar ribonucleoprotein), LocusLink
69. RNF30 ring finger protein 30, LocusLink=57159
70. PIG3 quinone oxidoreductase homolog, LocusLink=9540
71. MYT1L myelin transcription factor 1-like, LocusLink=23040
72. VIT1 vitiligo-associated protein VIT-1, LocusLink=55519
73. CAPG capping protein (actin filament), gelsolin-like, LocusLink=822
74. IL12A interleukin 12A (natural killer cell stimulatory factor 1, cytotoxic lymphocyte maturati
75. LOC51095 CGI-47 protein, LocusLink=51095
76. MRPS25 mitochondrial ribosomal protein S25, LocusLink=64432
77. GBE1 glucan (1,4-alpha-), branching enzyme 1 (glycogen branching enzyme, Andersen disease, gly
78. FETUB fetuin B, LocusLink=26998
79. SRP stresscopin-related peptide, LocusLink=90226
80. TCTA T-cell leukemia translocation altered gene, LocusLink=6988
81. JWA vitamin A responsive; cytoskeleton related, LocusLink=10550
82. ITIH1 inter-alpha (globulin) inhibitor, H1 polypeptide, LocusLink=3697
83. ATP1B3 ATPase, Na+/K+ transporting, beta 3 polypeptide, LocusLink=483
84. STAG1 stromal antigen 1, LocusLink=10274
85. LZTFL1 leucine zipper transcription factor-like 1, LocusLink=54585
86. RPL24 ribosomal protein L24, LocusLink=6152
87. WNT5A wingless-type MMTV integration site family, member 5A, LocusLink=7474
88. MLH1 mutL (E. coli) homolog 1 (colon cancer, nonpolyposis type 2), LocusLink=4292
89. B3GNT5 UDP-GlcNAc:betaGal beta-1,3-N-acetylglucosaminyltransferase 5, LocusLink=84002
90. LUZP3 leucine zipper protein 3, LocusLink=83598
91. MCCC1 methylcrotonoyl-Coenzyme A carboxylase 1 (alpha), LocusLink=56922
92. LOC51161 g20 protein, LocusLink=51161
93. NFKB1 nuclear factor of kappa light polypeptide gene enhancer in B-cells 1 (p105);
94. IRF2 interferon regulatory factor 2, LocusLink=3660
95. ARHH ras homolog gene family, member H, LocusLink=399
96. KSP37 Ksp37 protein, LocusLink=83888
97. MAP2K1IP1 mitogen-activated protein kinase kinase 1 interacting protein 1;
98. DDX15 DEAD/H (Asp-Glu-Ala-Asp/His) box polypeptide 15, LocusLink=1665
99. GRO1 GRO1 oncogene (melanoma growth stimulating activity, alpha), LocusLink=2919;
100. SCYB5 small inducible cytokine subfamily B (Cys-X-Cys), member 5 (epithelial-derived neutrophi
101. UBE2D3 ubiquitin-conjugating enzyme E2D 3 (homologous to yeast UBC4/5), LocusLink=732
102. GLRA3 glycine receptor, alpha 3, LocusLink=8001
103. EGF epidermal growth factor (beta-urogastrone), LocusLink=1950
104. RPL34 ribosomal protein L34 , LocusLink=6164
105. WHSC2 Wolf-Hirschhorn syndrome candidate 2, LocusLink=7469
106. RAB28 RAB28, member RAS oncogene family, LocusLink=9364
107. EREG epiregulin, LocusLink=2069
108. SEC3 Sec3-like, LocusLink=55763
109. SLC34A2 solute carrier family 34 (sodium phosphate), member 2, LocusLink=1
110. ZNF141 zinc finger protein 141 (clone pHZ-44), LocusLink=7700
111. NUP54 nucleoporin p54, LocusLink=53371
112. IL8 interleukin 8, LocusLink=3576
113. IL12B interleukin 12B (natural killer cell stimulatory factor 2, cytotoxic lymphocyte maturati
114. DCTN4 dynactin 4 (p62), LocusLink=51164
115. GPRK6 G protein-coupled receptor kinase 6, LocusLink=2870
116. OSMR oncostatin M receptor, LocusLink=9180
117. SLC1A3 solute carrier family 1 (glial high affinity glutamate transporter), member 3;
118. NAF1 Nef-associated factor 1, LocusLink=10318
119. P4HA2 procollagen-proline, 2-oxoglutarate 4-dioxygenase (proline 4-hydroxylase), alpha polypep
120. IL13 interleukin 13, LocusLink=3596
121. CSF2 colony stimulating factor 2 (granulocyte-macrophage), LocusLink=1437
122. NOLA2 nucleolar protein family A, member 2 (H/ACA small nucleolar RNPs), LocusLink=55
123. ODZ2 odd Oz/ten-m homolog 2 (Drosophila, mouse), LocusLink=57451
124. MRPS36 mitochondrial ribosomal protein S36, LocusLink=92259
125. PCDHGA11 protocadherin gamma subfamily A, 11, LocusLink=56105
126. TAF2F TATA box binding protein (TBP)-associated factor, RNA polymerase II, F, 55kD;
127. PRIM2A primase, polypeptide 2A (58kD), LocusLink=5558
128. H2BFA H2B histone family, member A, LocusLink=8339
129. BCKDHB branched chain keto acid dehydrogenase E1, beta polypeptide (maple syrup urine disease)
130. NDR serine threonine protein kinase, LocusLink=11329
131. RCL putative c-Myc-responsive, LocusLink=10591
132. NOTCH4 Notch (Drosophila) homolog 4, LocusLink=4855
133. LOC54516 similar to prokaryotic-type class I peptide chain release factors, LocusLink
134. PTD013 PTD013 protein, LocusLink=51389
135. SPR1 SPR1 protein, LocusLink=29112
136. TCF21 transcription factor 21, LocusLink=6943
137. G6E putative Ly-6 superfamily member, LocusLink=79136
138. EDN1 endothelin 1, LocusLink=1906
139. MICB MHC class I polypeptide-related sequence B, LocusLink=4277
140. BAT8 HLA-B associated transcript 8, LocusLink=10919
141. BAT4 HLA-B associated transcript 4, LocusLink=7918
142. HTR1B 5-hydroxytryptamine (serotonin) receptor 1B, LocusLink=3351
143. BF B-factor, properdin, LocusLink=629
144. SERPINB1 serine (or cysteine) proteinase inhibitor, clade B (ovalbumin), member 1;
145. DKFZP586D2223 DKFZP586D2223 protein, LocusLink=25862
146. ABT1 TATA-binding protein-binding protein, LocusLink=29777
147. DKFZP564B116 DKFZP564B116 protein, LocusLink=25998
148. CLIC5 chloride intracellular channel 5, LocusLink=53405
149. REV3L REV3 (yeast homolog)-like, catalytic subunit of DNA polymerase zeta;
150. STK31 serine/threonine kinase 31, LocusLink=56164
151. NOH61 putative nucleolar RNA helicase, LocusLink=54606
152. CUTL1 cut (Drosophila)-like 1 (CCAAT displacement protein), LocusLink=1523
153. CALN1 calneuron 1, LocusLink=83698
154. TFPI2 tissue factor pathway inhibitor 2, LocusLink=7980
155. TAC1 tachykinin, precursor 1 (substance K, substance P, neurokinin 1, neurokinin 2, neuromedin
156. LOC51024 CGI-135 protein, LocusLink=51024
157. HIC I-mfa domain-containing protein, LocusLink=29969
158. LKR/SDH lysine-ketoglutarate reductase /saccharopine dehydrogenase, LocusLink=10157;
159. PAX4 paired box gene 4, LocusLink=5078
160. ZNF212 zinc finger protein 212, LocusLink=7988
161. DFNA5 deafness, autosomal dominant 5, LocusLink=1687
162. AKR1D1 aldo-keto reductase family 1, member D1 (delta 4-3-ketosteroid-5-beta-reductase); chrom
163. NDUFB2 NADH dehydrogenase (ubiquinone) 1 beta subcomplex, 2 (8kD, AGGG);
164. NPD007 NPD007 protein, LocusLink=57414
165. EN2 engrailed homolog 2, LocusLink=2020
166. HTR5A 5-hydroxytryptamine (serotonin) receptor 5A, LocusLink=3361
167. TSLRP testis specific leucine rich repeat protein, LocusLink=23639
168. WISP1 WNT1 inducible signaling pathway protein 1, LocusLink=8840
169. FOG2 Friend of GATA2, LocusLink=23414
170. RPS20 ribosomal protein S20, LocusLink=6224
171. RAD21 RAD21 (S. pombe) homolog, LocusLink=5885
172. D8S2298E reproduction 8, LocusLink=7993
173. HT002 HT002 protein; hypertension-related calcium-regulated gene, LocusLink=28991
174. PNOC prepronociceptin, LocusLink=5368
175. DLGAP2 discs, large (Drosophila) homolog-associated protein 2, LocusLink=9228
176. DEFB2 defensin, beta 2, LocusLink=1673
177. INSL4 insulin-like 4 (placenta), LocusLink=3641
178. RGS3 regulator of G-protein signalling 3, LocusLink=5998
179. ROR2 receptor tyrosine kinase-like orphan receptor 2, LocusLink=4920
180. MTAP methylthioadenosine phosphorylase, LocusLink=4507
181. UNC13 UNC13 (C. elegans)-like, LocusLink=10497
182. C9orf9 chromosome 9 open reading frame 9, LocusLink=11092
183. DNAI1 dynein, axonemal, intermediate polypeptide, 1, LocusLink=27019
184. C9orf5 chromosome 9 open reading frame 5, LocusLink=23731
185. BRD3 bromodomain-containing 3, LocusLink=8019
186. CD72 CD72 antigen, LocusLink=971
187. TRAF1 TNF receptor-associated factor 1, LocusLink=7185
188. FPGS folylpolyglutamate synthase, LocusLink=2356
189. RXRA retinoid X receptor, alpha, LocusLink=6256
190. HSPC043 HSPC043 protein, LocusLink=58493
191. ZID zinc finger protein with interaction domain, LocusLink=10773
192. ENG endoglin (Osler-Rendu-Weber syndrome 1), LocusLink=2022
193. RENT2 regulator of nonsense transcripts 2, LocusLink=26019
194. NOLC1 nucleolar and coiled-body phosphprotein 1, LocusLink=9221
195. CSPG6 chondroitin sulfate proteoglycan 6 (bamacan), LocusLink=9126
196. FER1L3 fer-1 (C.elegans)-like 3 (myoferlin), LocusLink=26509
197. TIM23 translocase of inner mitochondrial membrane 23 (yeast) homolog, LocusLink=1043
198. PLA2G13 group XIII secreted phospholipase A2, LocusLink=84647
199. BUB3 BUB3 (budding uninhibited by benzimidazoles 3, yeast) homolog, LocusLink=918
200. PYCS pyrroline-5-carboxylate synthetase (glutamate gamma-semialdehyde synthetase)
201. CYP26A1 cytochrome P450, subfamily XXVIA, polypeptide 1, LocusLink=1592
202. NDUFB8 NADH dehydrogenase (ubiquinone) 1 beta subcomplex, 8 (19kD, ASHI)
203. TNFRSF6 tumor necrosis factor receptor superfamily, member 6, LocusLink=355
204. DEPP decidual protein induced by progesterone, LocusLink=11067
205. SEMA4G sema domain, immunoglobulin domain (Ig), transmembrane domain (TM) and short cytoplasmi
206. CPN1 carboxypeptidase N, polypeptide 1, 50kD, LocusLink=1369
207. BMI1 murine leukemia viral (bmi-1) oncogene homolog, LocusLink=648
208. PARG poly (ADP-ribose) glycohydrolase, LocusLink=8505
209. NUMA1 nuclear mitotic apparatus protein 1, LocusLink=4926
210. CHORDC1 cysteine and histidine-rich domain (CHORD)-containing, zinc-binding protein 1
211. FLI1 Friend leukemia virus integration 1, LocusLink=2313
212. MSP mosaic serine protease, LocusLink=84000
213. EEF1G eukaryotic translation elongation factor 1 gamma, LocusLink=1937
214. TMPRSS5 transmembrane protease, serine 5 (spinesin), LocusLink=80975
215. EHF ets homologous factor, LocusLink=26298
216. CLN2 ceroid-lipofuscinosis, neuronal 2, late infantile (Jansky-Bielschowsky disease)
217. SLC22A1L solute carrier family 22 (organic cation transporter), member 1-like
218. NDUFC2 NADH dehydrogenase (ubiquinone) 1, subcomplex unknown, 2 (14.5kD, B14.5b)
219. TRIM5 tripartite motif-containing 5, LocusLink=85363
220. KCNK7 potassium channel, subfamily K, member 7, LocusLink=10089
221. FEN1 flap structure-specific endonuclease 1, LocusLink=2237
222. VPS11 vacuolar protein sorting 11 (yeast homolog), LocusLink=55823
223. DLG2 discs, large (Drosophila) homolog 2 (chapsyn-110) (NOTE: redefinition of symbol)
224. APOC3 apolipoprotein C-III, LocusLink=345
225. APOA4 apolipoprotein A-IV, LocusLink=337
226. CASP4 caspase 4, apoptosis-related cysteine protease, LocusLink=837
227. SDHD succinate dehydrogenase complex, subunit D, integral membrane protein
228. MAP3K11 mitogen-activated protein kinase kinase kinase 11, LocusLink=4296
229. PME-1 protein phosphatase methylesterase-1, LocusLink=51400
230. HSMDPKIN myotonic dystrophy protein kinase like protein, LocusLink=55561
231. ASCL2 achaete-scute complex (Drosophila) homolog-like 2, LocusLink=430
232. COX8 cytochrome c oxidase subunit VIII, LocusLink=1351
233. UPK2 uroplakin 2, LocusLink=7379
234. TIMM8B translocase of inner mitochondrial membrane 8 (yeast) homolog B
235. BLR1 Burkitt lymphoma receptor 1, GTP-binding protein, LocusLink=643
236. SLC4A8 solute carrier family 4, sodium bicarbonate cotransporter, member 8
237. HOXC5 homeo box C5, LocusLink=3222
238. KCNA5 potassium voltage-gated channel, shaker-related subfamily, member 5
239. KCNA1 potassium voltage-gated channel, shaker-related subfamily, member 1 (episodic ataxia wit
240. GIT2 G protein-coupled receptor kinase-interactor 2, LocusLink=9815
241. IRAK-M interleukin-1 receptor-associated kinase M, LocusLink=11213
242. LOC51729 Npw38-binding protein NpwBP, LocusLink=51729
243. C2F putative protein, LocusLink=10436
244. ADMR adrenomedullin receptor, LocusLink=11318
245. AAAS achalasia, adrenocortical insufficiency, alacrimia (Allgrove, triple-A)
246. PTPRR protein tyrosine phosphatase, receptor type, R, LocusLink=5801
247. OAS1 2',5'-oligoadenylate synthetase 1 (40-46 kD), LocusLink=4938
248. LOC51290 CDA14, LocusLink=51290
249. PPFIBP1 PTPRF interacting protein, binding protein 1 (liprin beta 1), LocusLink=8496
250. IL23A interleukin 23, alpha subunit p19, LocusLink=51561
251. CRBPIII putative cellular retinol-binding protein CRBP III, LocusLink=83758
252. RFX4 regulatory factor X, 4 (influences HLA class II expression), LocusLink=5992
253. NCOR2 nuclear receptor co-repressor 2, LocusLink=9612
254. LUM lumican, LocusLink=4060
255. P11 protease, serine, 22, LocusLink=8909
256. UBE2N ubiquitin-conjugating enzyme E2N (homologous to yeast UBC13), LocusLink=7334;
257. AQP2 aquaporin 2 (collecting duct), LocusLink=359
258. RAB5B RAB5B, member RAS oncogene family, LocusLink=5869
259. TARBP2 TAR (HIV) RNA-binding protein 2, LocusLink=6895
260. MLL2 myeloid/lymphoid or mixed-lineage leukemia 2, LocusLink=8085
261. GALNT4 UDP-N-acetyl-alpha-D-galactosamine:polypeptide N-acetylgalactosaminyltransferase 4 (Gal
262. P85SPR PAK-interacting exchange factor beta, Locuslink=8874
263. HSP105B heat shock 105kD. LocusLink=10808
264. MAB21L1 mab-21 (c.elegans)-like 1, LocusLink=4081
265. RNF17 ring finger protein 17, LocusLink=56163
266. KCNK10 potassium channel, subfamily K, member 10 (TREK-2), LocusLink=54207
267. FOXG1B forkhead box G1B, LocusLink=2290
268. ADPRTL2 ADP-ribosyltransferase (NAD+; poly(ADP-ribose) polymerase)-like 2
269. TLH29 TLH29 protein precursor, LocusLink=83982
270. PLPL myelin proteolipid protein-like protein, LocusLink=56936
271. CEBPE CCAAT/enhancer binding protein (C/EBP), epsilon, LocusLink=1053
272. LOC85439 stonin 2, LocusLink=85439
273. CIDEB cell death-inducing DFFA-like effector b, LocusLink=27141
274. BATF basic leucine zipper transcription factor, ATF-like, LocusLink=10538
275. GTF2A1 general transcription factor IIA, 1 (37kD and 19kD subunits), LocusLink=2957;
276. EIF5 eukaryotic translation initiation factor 5, LocusLink=1983
277. PNN pinin, desmosome associated protein, LocusLink=5411
278. TINF2 TERF1 (TRF1)-interacting nuclear factor 2, LocusLink=26277
279. D15S226E Prader-Willi/Angelman syndrome-5, LocusLink=8123
280. LOC51049 clone 1900 unknown protein, LocusLink=51049
281. ISLR immunoglobulin superfamily containing leucine-rich repeat, LocusLink=367
282. CYP19 cytochrome P450, subfamily XIX (aromatization of androgens), LocusLink=1588
283. IGF1R insulin-like growth factor 1 receptor, LocusLink=3480
284. ITGA11 integrin, alpha 11, LocusLink=22801
285. HCN4 hyperpolarization activated cyclic nucleotide-gated potassium channel 4
286. MDS009 x 009 protein, LocusLink=56986
287. GTF2A2 general transcription factor IIA, 2 (12kD subunit), LocusLink=2958
288. MGC14386 similar to cyclin-E binding protein 1 (H. sapiens), LocusLink=91433
289. KNSL5 kinesin-like 5 (mitotic kinesin-like protein 1), LocusLink=9493
290. CRYM crystallin, mu, LocusLink=1428
291. PARD6A par-6 (partitioning defective 6, C.elegans) homolog alpha
292. ORC6L origin recognition complex, subunit 6 (yeast homolog)-like, LocusLink=23594; 2
293. SULT1A1 sulfotransferase family, cytosolic, 1A, phenol-preferring, member 1
294. RCD-8 autoantigen, LocusLink=23644
295. GNAO1 guanine nucleotide binding protein (G protein), alpha activating activity polypeptide O;
296. PLK polo (Drosophia)-like kinase, LocusLink=5347
297. HSPC171 HSPC171 protein, LocusLink=29100
298. PRO0461 PRO0461 protein, LocusLink=28993
299. GK001 GK001 protein, LocusLink=57003
300. TIP-1 Tax interaction protein 1, LocusLink=30851
301. SOX20 SRY (sex determining region Y)-box 20, LocusLink=6665
302. NAGLU N-acetylglucosaminidase, alpha- (Sanfilippo disease IIIB), LocusLink=46
303. HT008 uncharacterized hypothalamus protein HT008, LocusLink=55852
304. SLC4A1 solute carrier family 4, anion exchanger, member 1 (erythrocyte membrane protein band 3
305. RDGBB retinal degeneration B beta, LocusLink=26207
306. IFI35 interferon-induced protein 35, LocusLink=3430
307. AD023 AD023 protein, LocusLink=57409
308. USP22 ubiquitin specific protease 22, LocusLink=23326
309. LAK-4P expressed in activated T/LAK lymphocytes, LocusLink=11322
310. UBB ubiquitin B, LocusLink=7314
311. ARHGDIA Rho GDP dissociation inhibitor (GDI) alpha, LocusLink=396
312. VTS58635 ras-like protein VTS58635, LocusLink=91608
313. HSY11339 GalNAc alpha-2, 6-sialyltransferase I, long form, LocusLink=55808
314. MPP3 membrane protein, palmitoylated 3 (MAGUK p55 subfamily member 3)
315. HCNGP transcriptional regulator protein, LocusLink=29115
316. ZNF179 zinc finger protein 179, LocusLink=7732
317. HOXB4 homeo box B4, LocusLink=3214
318. HOXB2 homeo box B2, LocusLink=3212
319. SCYA23 small inducible cytokine subfamily A (Cys-Cys), member 23, LocusLink=636
320. NUP88 nucleoporin 88kD, LocusLink=4927
321. RECQL5 RecQ protein-like 5, LocusLink=9400
322. GALR2 galanin receptor 2, LocusLink=8811
323. KSR kinase suppressor of ras, LocusLink=8844
324. EPX eosinophil peroxidase, LocusLink=8288
325. SCYA11 small inducible cytokine subfamily A (Cys-Cys), member 11 (eotaxin)
326. AOC3 amine oxidase, copper containing 3 (vascular adhesion protein 1), LocusLink=
327. VATI membrane protein of cholinergic synaptic vesicles, LocusLink=10493
328. SOST sclerostin, LocusLink=50964
329. WNT3 wingless-type MMTV integration site family, member 3, LocusLink=7473
330. HML2 macrophage lectin 2 (calcium dependent), LocusLink=10462
331. GALK1 galactokinase 1, LocusLink=2584
332. SFRS2 splicing factor, arginine/serine-rich 2, LocusLink=6427
333. VTN vitronectin (serum spreading factor, somatomedin B, complement S-protein)
334. ZNF24 zink finger protein 24 (KOX17), LocusLink=84307
335. RNMT RNA (guanine –7-)methyltransferase, LocusLink=8731
336. MC2R melanocortin 2 receptor, LocusLink=4158
337. B29 B29protein, LocusLink=8876
338. BRUNOL4 Bruno (Drosophila)-like 4, RNA binding protein, LocusLink=56853
339. SNRPD1 smallnuclear ribonucleoprotein D1 polypeptide (16kD), LocusLink=6632
340. ELAC1 elaC (E.coli) homolog 1, LocusLink=55520
341. EDG5 endothelial differentiation, sphingolipid G-protein-coupled receptor, 5
342. GIOT-2 GIOT-2 for gonadotropin inducible transcription repressor-2, LocusLink=51710;
343. CD209L CD209 antigen-like, LocusLink=10332
344. SIRT6 sirtuin (silent mating type information regulation 2, S. cerevisiae, homolog) 6
345. SIRT2 sirtuin (silent mating type information regulation 2, S.cerevisiae, homolog) 2
346. CD37 CD37 antigen, LocusLink=951
347. AKT2 v-akt murine thymoma viral oncogene homolog 2, LocusLink=208
348. MCC2 AIE-75 binding protein protein, LocusLink=83878
349. CEACAM4 carcinoembryonic antigen-related cell adhesion molecule 4, LocusLink=10
350. CEACAM3 carcinoembryonic antigen-related cell adhesion molecule 3, LocusLink=10
351. CDC37 CDC37 (cell division cycle 37, S. cerevisiae, homolog), LocusLink=11140
352. COL5A3 collagen, type V, alpha 3, LocusLink=50509
353. CYP4F11 cytochrome P450, subfamily IVF, polypeptide 11, LocusLink=57834
354. IRF3 interferon regulatory factor 3, LocusLink=3661
355. BC-2 putative breast adenocarcinoma marker (32kD), LocusLink=27243
356. TNFSF7 tumor necrosis factor (ligand) superfamily, member 7, LocusLink=970
357. NSP1 novel SH2-containing protein 1, LocusLink=10045
358. CYP4F2 cytochrome P450, subfamily IVF, polypeptide 2, LocusLink=8529
359. EEF2 eukaryotic translation elongation factor 2, LocusLink=1938
360. BBC3 Bcl-2 binding component 3, LocusLink=27113
361. APOC1 apolipoprotein C-I, LocusLink=341
362. LOC51231 VRK3 for vaccinia related kinase 3, LocusLink=51231
363. INSR insulin receptor, LocusLink=3643
364. EZFIT endothelial zinc finger protein induced by tumor necrosis factor alpha
365. KLK4 kallikrein 4 (prostase, enamel matrix, prostate), LocusLink=9622
366. RELB v-rel avian reticuloendotheliosis viral oncogene homolog B (nuclear factor of kappa light
367. KLK1 kallikrein 1, renal/pancreas/salivary, LocusLink=3816
368. ACP5 acid phosphatase 5, tartrate resistant, LocusLink=54
369. LOC91120 similar to ZINC FINGER PROTEIN 85 (ZINC FINGER PROTEIN HPF4) (HTF1) (H. sapiens); chr
370. ZNF136 zinc finger protein 136 (clone pHZ-20), LocusLink=7695
371. TZFP testis zinc finger protein, LocusLink=27033
372. ICAM3 intercellular adhesion molecule 3, LocusLink=3385
373. ICAM1 intercellular adhesion molecule 1 (CD54), human rhinovirus receptor
374. LILRB5 leukocyte immunoglobulin-like receptor, subfamily B (with TM and ITIM domains), member
375. LILRB4 leukocyte immunoglobulin-like receptor, subfamily B (with TM and ITIM domains), member
376. LILRB3 leukocyte immunoglobulin-like receptor, subfamily B (with TM and ITIM domains), member
377. PNKP polynucleotide kinase 3'-phosphatase, LocusLink=11284
378. GP6 glycoprotein VI (platelet), LocusLink=51206
379. ZNF226 zinc finger protein 226, LocusLink=7769
380. ZNF222 zinc finger protein 222, LocusLink=7673
381. PPP1R15A protein phosphatase 1, regulatory (inhibitor) subunit 15A, LocusLink=2
382. NFKBIB nuclear factor of kappa light polypeptide gene enhancer in B-cells inhibitor, beta; chr
383. LENG4 leukocyte receptor cluster (LRC) member 4, LocusLink=79143
384. GPR4 G protein-coupled receptor 4, LocusLink=2828
385. PMP24 24 kDa intrinsic membrane protein, LocusLink=11264
386. CHGB chromogranin B (secretogranin 1), LocusLink=1114
387. GMEB2 glucocorticoid modulatory element binding protein 2, LocusLink=26205
388. DPM1 dolichyl-phosphate mannosyltransferase polypeptide 1, catalytic subunit
389. LOC51605 CGI-09 protein; , LocusLink=51605
390. SGK2 serum/glucocorticoid regulated kinase 2; , LocusLink=10110
391. PTPN1 protein tyrosine phosphatase, non-receptor type 1, LocusLink=5770;
392. ADF destrin (actin depolymerizing factor), LocusLink=11034
393. RPC39 polymerase (RNA) III (DNA directed) (39kD), LocusLink=10621
394. ARFRP1 ADP-ribosylation factor related protein 1, LocusLink=10139
395. TUBB1 beta tubulin 1, class VI, LocusLink=81027
396. DLM1 tumor stroma and activated macrophage protein DLM-1 , LocusLink=81030
397. FLJ22504 hypothetical C2H2 zinc finger protein FLJ22504 , LocusLink=63925
398. CLG01 clg01 protein , LocusLink=27296
399. LOC55902 acetyl-CoA synthetase; , LocusLink=55902 TMPRSS3 transmembrane rpotease, serine 3, LocusLink=64699
400. CRYAA crystallin alpha A, LocusLink=1409
401. COL6A2 collagen, type VI, alpha2, LocusLink=1292
402. CBR1 carbonyl reductase 1, LocusLink=873
403. ATP50 ATP synthase, H^+^ transporting, mitochndrial F1 complex, O subunit
404. GALR3 galanin receptor 3, LocusLink=8484
405. PLA2G6 phospholipase A2, group VI (cytosolic, calcium-independent), LocusLink=8
406. RFPL2 ret finger protein-like 2, LocusLink=10739
407. RFPL1 ret finger protein-like 1, LocusLink=5988
408. CTRP6 complement-c1q tumor necrosis factor-related protein 6, LocusLink=83847
409. TCN2 transcobalamin II; macrocytic anemia, LocusLink=6948
410. C22orf5 chromosome 22 open reading frame 5, LocusLink=25829
411. EIF3S7 eukaryotic translation initiation factor 3, subunit 7 (zeta, 66/67kD)
412. PPARA peroxisome proliferative activated receptor, alpha, LocusLink=5465; 2
413. DNAL4 dynein, axonemal, light polypeptide 4, LocusLink=10126
414. RBM9 RNA binding motif protein 9, LocusLink=23543
415. VPREB3 pre-B lymphocyte gene 3, LocusLink=29802
416. ARSE arylsulfatase E (chondrodysplasia punctata 1), LocusLink=415
417. SOX3 SRY (sex determining region Y)-box 3, LocusLink=6658
418. RBM10 RNA binding motif protein 10, LocusLink=8241
419. RPS4X ribosomal protein S4, X-linked, LocusLink=6191
420. APR-1 APR-1 protein, LocusLink=28986
421. LOC51634 CGI-79 protein, LocusLink=51634
422. APEXL2 apurinic/apyrimidinic endonuclease(APEX nuclease)-like 2 protein, LocusLink=27
423. RPL44 ribosomal protein L44, LocusLink=6173
424. FLJ20494 similar to mouse neuronal protein 15.6, LocusLink=54539
425. SLC35A2 solute carrier family 35 (UDP-galactose transporter), member 2
426. IKBKG inhibitor of kappa light polypeptide gene enhancer in B-cells, kinase gamma
427. ARAF1 v-raf murine sarcoma 3611 viral oncogene homolog 1, LocusLink=369;
428. ELK1 ELK1, member of ETS oncogene family, LocusLink=2002
429. SLC6A14 solute carrier family 6 (neurotransmitter transporter), member 14
430. RBMY1A1 RNA binding motif protein
